# Application of the Phylogenetic Species Concept to *Wallemia sebi* from House Dust and Indoor Air Revealed by Multi-Locus Genealogical Concordance

**DOI:** 10.1371/journal.pone.0120894

**Published:** 2015-03-23

**Authors:** Hai D. T. Nguyen, Sašo Jančič, Martin Meijer, Joey B. Tanney, Polona Zalar, Nina Gunde-Cimerman, Keith A. Seifert

**Affiliations:** 1 Department of Biology, Faculty of Science, University of Ottawa, Ottawa, Ontario, Canada; 2 Biodiversity (Mycology), Eastern Cereal and Oilseed Research Centre, Agriculture and Agri-Food Canada, Ottawa, Ontario, Canada; 3 Department of Biology, Biotechnical Faculty, University of Ljubljana, Ljubljana, Slovenia; 4 CBS-KNAW Fungal Biodiversity Centre, Utrecht, The Netherlands; Consiglio Nazionale delle Ricerche (CNR), ITALY

## Abstract

A worldwide survey of *Wallemia* occurring in house dust and indoor air was conducted. The isolated strains were identified as *W*. *sebi* and *W*. *muriae*. Previous studies suggested that the *W*. *sebi* phylogenetic clade contained cryptic species but conclusive evidence was lacking because only the internal transcribed spacer (ITS) marker was analyzed. The ITS and four protein-coding genes (*MCM*7, *RPB*1, *RPB*2, and *TSR*1) were sequenced for 85 isolates. Based on an initial neighbor joining analysis of the concatenated genes, *W*. *muriae* remained monophyletic but four clades were found in *W*. *sebi*, which we designated as *W*. *sebi* clades 1, 2, 3, and 4. We hypothesized that these clades represent distinct phylogenetic species within the *Wallemia sebi* species complex (WSSC). We then conducted multiple phylogenetic analyses and demonstrated genealogical concordance, which supports the existence of four phylogenetic species within the WSSC. Geographically, *W*. *muriae* was only found in Europe, *W*. *sebi* clade 3 was only found in Canada, *W*. *sebi* clade 4 was found in subtropical regions, while *W*. *sebi* clade 1 and 2 were found worldwide. Haplotype analysis showed that *W*. *sebi* clades 1 and 2 had multiple haplotypes while *W*. *sebi* clades 3 and 4 had one haplotype and may have been under sampled. We describe *W*. *sebi* clades 2, 3, and 4 as new species in a companion study.

## Introduction

The genus *Wallemia* was introduced over a century ago by Johan-Olsen [1] for *W*. *ichthyophaga*, discovered on dried salted fish. However, *Wallemia* remained obscure and what are now recognized as *Wallemia* species were reported under several generic names. Von Arx [2] proposed the combination *W*. *sebi* for the species originally described as *Sporendonema sebi* Fr. and today it is the most frequently reported *Wallemia* species. In a recent study, some species names used in the old literature for *Wallemia* were synonymized and other doubtful names were listed as synonyms of *W*. *sebi* [3]. That study connected the old literature with modern concepts of *Wallemia* with molecular data [3]. As a consequence, three species, namely *W*. *sebi*, *W*. *muriae*, and *W*. *ichthyophaga*, were now recognized and grouped in the newly erected class Wallemiomycetes and order Wallemiales [3]. A more detailed review of the taxonomic history of *Wallemia* and its species is provided in the accompanying paper [4].

*Wallemia* was considered an enigma in the fungal kingdom and its taxonomic position remained uncertain for over a hundred years. Terracina [5] showed dolipore-like septal structures in *W*. *sebi*, similar to those formed by many fungi in the Basidiomycota and some yeasts in the Ascomycota. A few decades later, Moore [6] interpreted this as a special kind of parenthesome and described a new family, the Wallemiaceae, to accommodate *Wallemia*. The Wallemiaceae was first classified in the Filobasidiales (Basidiomycota). Subsequent studies could not confirm the exact evolutionary position of *Wallemia* within Basidiomycota by phylogenetic analysis with ribosomal DNA sequences and a few protein-coding gene sequences [7]. Recently, the genome of *W*. *sebi* was sequenced and a phylogenomic analysis with 71 protein-coding genes showed clearly that *Wallemia* belonged to a lineage basal to the Agaricomycotina (Basidiomycota) [8].

Morphologically, *Wallemia* species grow as powdery, brown colonies on low water activity media and are considered xerophilic or at least xerotolerant. The spore ontogenesis of this fungus is unusual and was the focus of many studies [8–11] because mycologists were undecided on whether *Wallemia* produces asexual or sexual spores. *Wallemia sebi* produces chains of blastic conidia that mature in basipetal succession by differentiation of a basauxically developing fertile hypha [12]. The elongating fertile hypha undergoes septation and subdivides into four cylindrical cells that swell and then disarticulate, a process that is reminiscent of thallic ontogeny. Recently, nuclear behavior during spore development was observed using differential interference contrast and epifluorescence microscopy [8]. Researchers reported no evidence of meiosis, concluding that the known morphology of this fungus represents an asexual morph [8]. Although the sexual morph of *Wallemia* has never been observed, a mating type locus and meiotic genes were detected in the genome of *W*. *sebi* CBS 633.66 [8]. Distantly related to *W*. *sebi* CBS 633.66, *Wallemia ichthyophaga* EXF-994 lacks a complete set of core meiosis genes and it might be incapable of sexual reproduction [13]. Thus, some *Wallemia* species may be capable of sexual reproduction but their sexual morphs remain undiscovered.

Many fungi exhibit cryptic speciation. A single morphological or biological species with a cosmopolitan distribution is often composed of multiple cryptic, phylogenetic species that are often geographically separated [14]. Sequence variation in the rDNA internal transcribed spacers region (ITS, i.e. ITS1-5.8S-ITS2) hints at the existence of cryptic species within *W*. *sebi* and this was noted previously [3]. Although ITS is the formally recognized fungal barcode [15], it sometimes does not distinguish among closely related phylogenetic species. The genealogical concordance phylogenetic species recognition concept (GCPSR) was proposed as an empirical method for recognizing cryptic speciation [16]. GCPSR involves sequencing multiple genes that are then combined in phylogenetic analyses. Incongruent nodes are identified as the point of genetic isolation and therefore the species limit (see [17] for *Xanthoparmelia*; [18] for *Penicillium*; [14] for *Neurospora*; [19] for *Fusarium*). The GCPSR is especially practical for delimiting species in morphologically reduced fungi or fungi that only exhibit their asexual morph like *Wallemia*.

Ecologically, *Wallemia* is a ubiquitous genus that is usually isolated from xeric environments, including sweet (fruits, jams, cakes) and salty (fish, bacon, salted beans) foods, soil, hypersaline water of salterns [3], [20], pollen baskets and plants (Jančič et al. unpublished). In rare cases, *W*. *sebi* causes subcutaneous phaeohyphomycosis [21–25]. Chronic exposure to mould is often associated with allergy and asthma (reviewed in [26]). Sensitization to *W*. *sebi* was first reported in Japan [27] and another study showed that 0.2% of 1790 children aged 3–14 in Germany had IgE sensitization to *W*. *sebi* [28]. Occupational allergy to *W*. *sebi* was also reported in European farmers [29–32] as a condition called farmer’s lung disease, which is characterized by the inflammation of the lungs caused by inhalation of dust from mouldy hay or grain. It was reported recently that human antibodies react to compounds produced by *W*. *sebi* spores [33].

*Wallemia sebi* and *W*. *muriae* are the two species of *Wallemia* most commonly isolated from the indoor environment, an arid niche where xerophiles are common [3], [34–39]. *Wallemia sebi* was frequently isolated from house dust [27], [38] and detected by 454 pyrosequencing of house dust in Canada, USA, and Western Europe [40], [41]. At the same time as our metagenomic study [41], a parallel project was initiated to investigate the fungal biodiversity of the same samples using high throughput dilution-to-extinction culturing methods. The current study is part of that project. Here, we combined indoor *Wallemia* strains from two other studies that used air and swab sampling as isolation methods, to increase our sample size and geographic coverage. For reference, we included ex-neotype strains of *W*. *sebi* and *W*. *muriae*, and the genome sequenced strain of *W*. *sebi* (CBS 633.66). Our first objective was to identify what *Wallemia* species occurred in the indoor environment. Our second objective was to develop additional DNA markers to apply the GCPSR to delimit putative cryptic species in the *W*. *sebi* species complex (WSSC). We chose two protein-coding genes, RNA polymerase II largest subunit (*RPB*1) and RNA polymerase II second largest subunit (*RPB*2) that were previously used to separate species in the Basidiomycota [42–44]. Additionally, we selected two other genes, DNA replication licensing factor (*MCM*7) and pre-rRNA processing protein (*TSR*1), both recently identified as reliable markers for fungal molecular phylogenetics [45], [46]. After sequencing all five genes for all our isolates, we performed single gene and combined gene phylogenetic analyses. As a third objective, we analyzed two *W*. *sebi* strains reported to cause skin lesions [25] and a strain of indoor *W*. *sebi* reported to produce compounds that react to human antibodies [33] with our indoor strains to determine whether potentially medically relevant phylogenetic species exist in the WSSC.

This study establishes four DNA markers not previously used for *Wallemia* to detect cryptic speciation in the WSSC. The observed clades in the WSSC are taxonomically described as new species in a companion study [4], where physiological and secondary metabolite profiling are applied as phenotypic tests of the phylogenetic species hypotheses derived here.

## Materials and Methods

### Sample collection, isolation and culture

House dust samples were collected as previously described [41]. Briefly, sterilized dust stream collectors (Indoor Biotechnologies) were attached to domestic vacuum cleaners. Samples were collected through a 2-mm sieve and refrigerated at 4°C until further processing. For house dust, cultures were isolated by a modified dilution-to-extinction plating technique of house dust [47]. Air samples of 100 L were collected approximately 1 m above the ground with a viable impaction sampler (Sas Super ISO, PBI International). Indoor surfaces (ie. walls, ceilings) were sampled with the swab (Heinz Herenz, Hamburg, Germany). For air and swab sampling, cultures were isolated using standard microbiological techniques. Media for xerophilic fungi were used for isolation, such as malt extract agar with 20% sucrose (M20S: 20 g Bacto malt extract (Difco Laboratories, Sparks, USA); 200 g sucrose (EMD Chemicals Inc., Gibbstown, USA); 15 g agar (EMD Chemicals Inc., Gibbstown, USA); 1000 mL distilled water), malt and yeast extract with 40% sucrose (M40Y: 20 g Bacto malt extract (Difco Laboratories, Sparks, USA); 5 g Bacto yeast extract (Difco Laboratories, Sparks, USA); 400 g of sucrose (EMD Chemicals Inc., Gibbstown, USA); 15 g agar (EMD Chemicals Inc., Gibbstown, USA); 1000 mL distilled water), or dichloran 18% glycerol (DG18: Oxoid Ltd, Hampshire, UK) agar and incubated at room temperature and inspected regularly. Putative *Wallemia* colonies were morphologically identified using a light microscope, transferred to M20S, and then transferred to M40Y prior to long-term preservation. Cultures were deposited and maintained at the Canadian Collection of Fungal Cultures, Agriculture and Agri-Food Canada (CCFC/DAOM), in Ottawa, Canada; CBS-KNAW Fungal Biodiversity Centre, Utrecht, the Netherlands (CBS); and the Ex Culture Collection of the Department of Biology, Biotechnical Faculty, University of Ljubljana, Infrastructural Centre Mycosmo, MRIC UL, Ljubljana, Slovenia (EXF). S1 Table includes information on all strains used in this study.

### Genetic marker development and evaluation

*Wallemia sebi* specific primers were designed using PrimaClade [48] for *RPB*1 and *RPB*2 genes from MAFFT v7.122b [49] alignment of existing *Wallemia* sequences [7]. *Wallemia sebi* specific primers for *MCM*7 and *TSR*1 genes were designed from the genome annotations of the *W*. *sebi* CBS 633.66 [8] using Primer3 [50], [51]. Markers were checked by BLAST against the *W*. *sebi* CBS 633.66 genome to verify that they were single copy and could be assumed to be unlinked because they are located on different scaffolds. All primer sequences used are shown in S2 Table.

### DNA extraction, PCR and sequencing

DNA extraction, PCR and sequencing were performed using a previously described method [52]. The following PCR profile was used to amplify ITS, *MCM*7, *RPB*1, and *TSR*1: 95°C for 3 min (initial denaturation), then 40 cycles at 95°C for 30 sec (denaturation), 55°C for 30 sec (annealing), 72°C for 1 min (extension), followed by 72°C for 5 min (final extension). A touchdown PCR profile was used to amplify *RPB*2. This profile was the same as the profile described above except that the annealing temperature started at 65°C (1 cycle), then changed to 63°C (1 cycle), then to 61°C (1 cycle), then to 59°C (1 cycle) then finally to 57°C (35 cycles).

### Clade assignment and phylogenetic analysis

Sequences of each gene were aligned using MAFFT v7.122b [49] with option L-INS-i for ITS and G-INS-i for *MCM*7, *TSR*1, *RPB*1, and *RPB*2. Alignments were trimmed with BioEdit v7.2.2 [53] and analyzed as described below.

To initially assess whether strains formed distinct phylogenetic clusters, a preliminary neighbor joining (NJ) analysis was performed on a concatenated data set of all aligned genes using SeaView v4.4.2 [54] with the following options: NJ; observed distance; do not ignore all gap sites.

Next, individual genes were analyzed using four methods: neighbor joining, maximum parsimony, maximum likelihood and Bayesian inference. NJ was performed in SeaView v4.4.2 [54] as described above with 1000 bootstrap replicates. Maximum parsimony heuristic searches were performed using PAUP4.10b [55] with these parameters: uninformative characters excluded, midpoint rooting, simple sequence addition, TBR swapping algorithm, collapse and multitrees in effect, 100 maximum trees saved. This was followed by the computation of a parsimony strict consensus tree. RAxML 8.0.20 [56] was used to compute a maximum likelihood tree using the GTRGAMMA model, chosen because it includes the parameter G for rate heterogeneity among sites. In RAxML, by default, G has 25 rate categories making the estimation of proportion of invariable sites (I) unnecessary because G mathematically accounts for I [57]. Support values were assessed using the ‘rapid bootstrapping’ option with 1000 replicates. Prior to Bayesian inference, jmodeltest v2.1.4 [58], [59] was used to calculate the best evolutionary model for each gene; for each gene alignment, likelihood scores were computed with the following options: 3 substitution schemes, base frequencies on (+F); rate variation on with 8 rate categories (+G, nCat = 8); ML optimized base tree; NNI search algorithm. The proportion of invariable sites (+I) was not considered in our model testing because it had minimal impact on estimates of rates and coalescence times for closely related species [60]. The HKY + G model was selected for ITS, *RPB*2 and *TSR*1 loci, and K80 + G was chosen for *MCM*7 and *RPB*1, according to the Bayesian Information Criterion (BIC) [61]. Bayesian inference analyses were performed with BEAST v2.1.3 [62]. BEAUTi v2.1.3 was used to generate the input XML file. Gene alignments were loaded in BEAUTi and each gene partition was assigned a separate site model, clock model and tree model. The site model was chosen according to the results from jmodeltest described above and the gamma category count was set to 8. All substitution rates, the gamma shape, and the kappa parameter were estimated and left on default settings. All of our *Wallemia* strains were closely related, so we chose the estimated strict clock and the Yule model of speciation, which does not take into account species extinction, conditional on the root for all gene partitions. The birth rate, clock rate and mutation rate priors were set to exponential, except the mutation rate for *RPB2* was set to uniform. Kappa parameters for the HKY models were left on lognormal. Then, the MCMC chain length was set to 1.0 x 10^8^ and storing one tree every 20000 generations. Three independent BEAST experiments were run with a different random seed. Convergence and effective sample size was monitored with Tracer v1.6. All gene trees from each independent run were combined with LogCombiner v2.1.3 with a burn-in of 10%. The consensus tree was generated with TreeAnnotator v2.1.3 with the target tree type set to maximum clade credibility tree and node heights set to mean heights.

All trees generated from these analyses (S1 File) were imported into FigTree v1.3.1 (http://tree.bio.ed.ac.uk/software/figtree/). Isolates were assigned to a clade number if they were recovered as a distinct group in the strict parsimony analyses and with >80% support values in the NJ, maximum likelihood and Bayesian analyses. We started the assessment on the right hand side of the tree (most recent in molecular time) and worked to the left, using groupings in the initial NJ tree based on the concatenated alignment.

After the isolates were assigned to clades, we used the species phylogeny approach by [63] implemented in *BEAST. *BEAST infers a species tree by considering divergence times, population sizes, and gene trees from multiple genes sampled from multiple individuals using a mixture of coalescent and Yule processes. Alignments were imported into the *BEAST template inside BEAUTi. We used the same setup parameters as for the Bayesian analysis described above for the site models, clock models and priors. Additionally, the Yule model conditional on the root was chosen for the species tree branching prior, the species birthrate and the population mean prior distributions were set to normal. Each strain was designated as a separate species using a mapping tab delimited file. Isolates that lacked sequence information for certain genes were included but the missing sequences were filled in with “?”, treated by BEAST as missing information. As above, the MCMC chain length was set to 1.0 x 10^8^ and storing one tree every 20000 generations. A total of 3 independent *BEAST experiments were run with a different random seed. Convergence and effective sample size was monitored with Tracer v1.6. The species trees from all independent runs were combined with LogCombiner v2.1.3 with a burn-in of 25%. The consensus species tree was generated with TreeAnnotator v2.1.3 with the target tree type set to maximum clade credibility tree and node heights set to mean heights.

To provide stronger support for the species hypothesis, a species delimitation analysis was conducted using the program BPP3 [64], [65], which uses a Bayesian approach to evaluate species delimitation. We used the preliminary NJ tree from the concatenated data set of all aligned genes described above as a guide tree. This method accommodates the species phylogeny as well as incomplete lineage sorting caused by ancestral polymorphism. A gamma prior *G*(2, 1000), with mean 2/2000 = 0.001, is used on the population size parameters (θs). The age of the root in the species tree (τ0) is assigned the gamma prior *G*(2, 1000), while the other divergence time parameters are assigned the Dirichlet prior [65]. Each analysis was run three times to confirm consistency between runs.

To compare the resolution of these markers as potential secondary barcodes, MEGA 5 [66] was used to calculate uncorrected pair wise distances (p-distance) between each sequence for each gene. This information was used to calculate the between clades and within clades p-distances using Microsoft Excel.

All sequences were deposited in GenBank (S1 Table). Alignments and trees were deposited in TreeBASE under study accession no. S15232.

### Haplotype analysis and geography

The program COLLAPSE v1.2 [67] was used to determine the haplotypes (i.e. unique sequences) in each gene alignment. We obtained a sequence of all strains for *MCM*7, *RPB*1 and *TSR*1. Missing data may have a negligible effect on species tree reconstruction [68], but haplotyping using DNA sequences would be sensitive to missing data; therefore our incomplete *RPB*2 *and ITS* data sets were excluded., Alignments were further trimmed with BioEdit v7.2.2 to eliminate all columns with missing data, then concatenated in SeaView v4.4.2 [54]. Following this, COLLAPSE v1.2 was run with default settings (gaps treated as 5^th^ state; sequences with 0 difference collapsed) to calculate the number of haplotypes.

### Ethics statement

The dust samples used in this study were collected from public or privately owned buildings by the owners or occupants of those buildings, with the informed consent of those individuals that fungal cultures would be isolated. Although applications were filed to approve collection and cross-border shipments, permits were not required for house dust. Similarly, permits requirements for living cultures of *Wallemia* imported into Canada were waived by the Canadian Food Inspection Agency. The *Wallemia* strains originating from Germany were obtained as part of a laboratory certification process in which indoor fungal isolates are distributed each year to test identification proficiency. No further data about these strains other than the city are available and they are considered publicly available cultures for research purposes. Previously studied *Wallemia* strains are cited in S1 Table. No protected lands were accessed and no protected species were sampled in this study.

## Results

### Isolates

A total of 85 isolates of *Wallemia* were isolated from our survey of house dust or indoor air in 12 countries: 22 strains from Slovenia; 15 from the Netherlands; 10 from the Federation of Micronesia; 7 from Germany; 6 from Denmark; 6 from Uruguay; 5 from Indonesia; 4 from Canada; 4 from United Kingdom; 3 from Thailand; 2 from Mexico; and 1 from South Africa. The source and method of isolation for each strain are summarized in S1 Table.

### Genetic marker assessment

For *Wallemia*, the ITS region amplifies easily but has a high sequencing failure rate. ITS sequence chromatograms often had multiple different overlapping peaks. We attempted to design *Wallemia* specific primers for the ITS region, but after pilot testing they were no more reliable for sequencing than standard primers [69]. Finally, with much difficulty, we were able to obtain ITS sequences for 70 of 90 strains. Even with 78% completion of the data from our indoor *Wallemia* strains, we were able to confirm the observation of potentially cryptic species within the WSSC [3].

To demonstrate cryptic speciation within the WSSC using GCPSR, we designed primers to amplify other markers. We designed primers for the genes *MCM*7, *RPB*1, *RPB*2, and *TSR*1 yielding amplicons of 603, 610, 738, and 607 bp respectively (Table 1). Our primers for *MCM*7, *RPB*1, and *TSR*1 yielded 100% sequencing success, while those for *RPB*2 were successful for 85 of 90 (94%) of the strains.

**Table 1:** Genetic variability of sampled loci.

Locus	# sequences	# unique sequences	Aligned bp	# alignment patterns^a^	# parsimony informative sites^b^	model^c^
ITS	70	50	513	123	39	HKY+G
*MCM*7	90	57	603	127	74	K80+G
*RPB*1	90	37	610	131	74	K80+G
*RPB*2	85	45	738	89	60	HKY+G
*TSR*1	90	49	607	128	69	HKY+G
Combined	90	NA	3071	598	316	NA

NA = not applicable

^a^RAxML v8.0.20 [56] detected the number of alignment patterns

^b^Number of parsimony informative sites were determined by PAUP v4.10b [55]

^c^The best model of sequence evolution was found by running jmodeltest v2.1.4 [58], [59]

**Fig 1:**
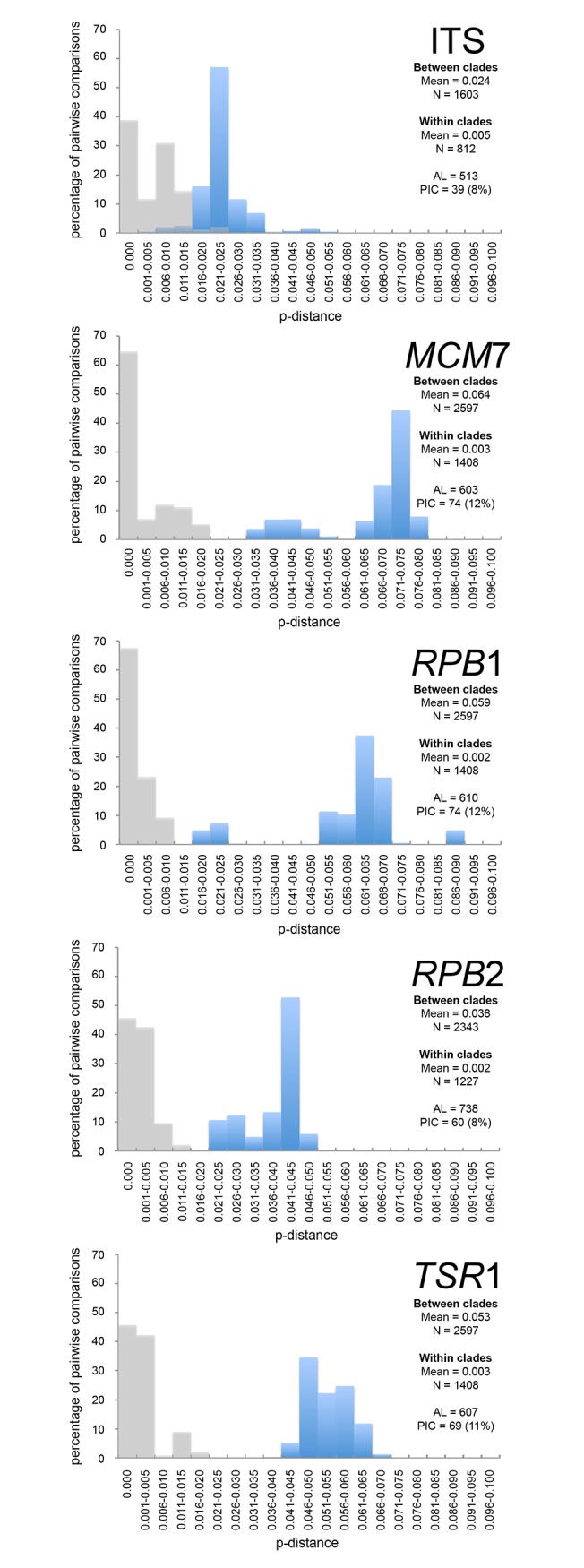
Comparison of pair-wise distance (p-distance), alignment length, and parsimony informative characters of each gene. The grey bars show the distribution range of the p-distance within clades while the blue bars show the range of p-distances between clades. The mean p-distances and the number of observations (N) used to calculate each mean are shown. AL = alignment length in base pairs. PIC = number and percentage of parsimony informative characters in the alignment.

To compare the resolution of these markers as potential secondary barcodes, we calculated the pairwise distance (p-distance) between all sequences. We then organized these values into two groups: the p-distances obtained from between clade comparisons and those obtained from within clade comparisons according to our species hypothesis. Ranges were graphed for each marker (Fig. 1). *MCM*7, *TSR*1, and *RPB*1 had high percentages of informative characters per sequenced base (11–12%), while ITS and *RPB*2 were lower at 8%. Additionally, *MCM*7, *TSR*1, and *RPB*1 showed a high mean between clade p-distance (0.053–0.064) while retaining low mean within clade p-distance (0.002–0.003). *RPB*2 had a lower mean between clade p-distance of 0.038 and a comparable mean within clade p-distance of 0.002. Meanwhile, ITS showed the lowest mean between clades p-distance (0.024) while having the highest mean within clades p-distance (0.005). We observed that the within clade and between clades p-distances overlapped for ITS, while these did not overlap for *MCM*7, *RPB*1, *RPB*2, and *TSR*1.

### Phylogenetic analysis

The initial neighbor joining (NJ) analysis with concatenated gene sequences revealed four distinct clades near the *W*. *sebi* neotype, comprising what we call the *W*. *sebi* complex (WSSC). We provisionally named them *W*. *sebi* clades 1, 2, 3, and 4. The isolates that made up clade 1 clustered around the ex-neotype strain of *W*. *sebi* (CBS 818.96). Those comprising clade 2 grouped with the genome sequenced strain of *W*. *sebi* (CBS 633.66). Clade 3 and 4 were not detected in previously [3] and did not group with any strains previously analyzed. The *W*. *muriae* strains grouped in the clade including the ex-neotype strain of *W*. *muriae* (CBS 116628).

**Fig 2:**
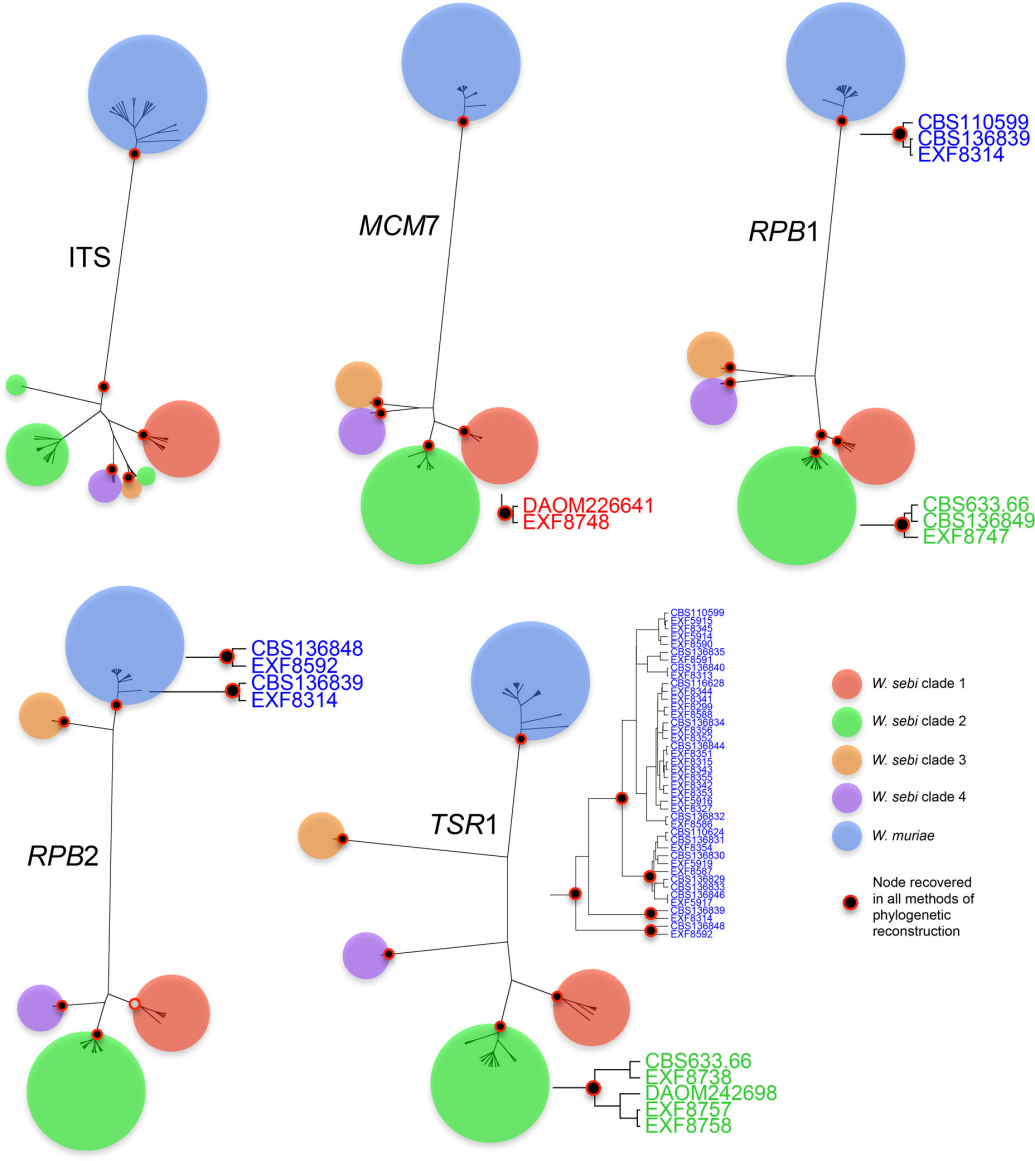
Single gene phylogenies of ITS, *MCM*7, *RPB*1, *RPB*2 and *TSR*1. The star trees topologies from the Bayesian analyses are illustrated. The black dot shows recovered distinct groups in the strict parsimony analyses and with >80% support values the NJ, maximum likelihood and Bayesian analyses. The grey dot in the *RPB*2 phylogeny marks the node supported by all analyses but with low bootstrap support in maximum likelihood. In each gene, certain nodes internal to our species hypothesis were well supported and these clades are depicted beside each gene tree.

We formulated species hypotheses based on this initial NJ analysis and designated nodes delineating monophyletic groups, numbered as above, with *W*. *muriae* as the species limit. To support these hypotheses, genealogical concordance and monophyly must be demonstrated consistently across multiple loci. We performed single gene phylogenetic analyses with four different methods of phylogenetic reconstruction: NJ, parsimony, maximum likelihood and Bayesian inference. The support values for nodes in each single gene phylogeny are summarized in S4 Table and Fig. 2. Concordance for our designated clades was found for all single gene analyses with all four methods of reconstruction, with a few exceptions. The most obvious exception was found in the phylogeny of the ITS locus, where *W*. *sebi* clade 2 was polytomous instead of monophyletic, as found in the phylogeny of the other four loci. The second exception was that *W*. *sebi* clade 1 had a low bootstrap support value (51%), but only in the maximum likelihood analysis of the *RPB*2 locus.

*MCM*7, *RPB*1, *RPB*2, and *TSR*1 had a higher sequencing success rate than ITS and could easily distinguish *W*. *sebi* clades 1, 2, 3, and 4. However, as shown in the phylogenies (S1 File), ITS sequences can still recognize *W*. *muriae* and *W*. *sebi* clades 1, 3, and 4 but cannot distinguish *W*. *sebi* clade 2 as a monophyletic group.

**Fig 3:**
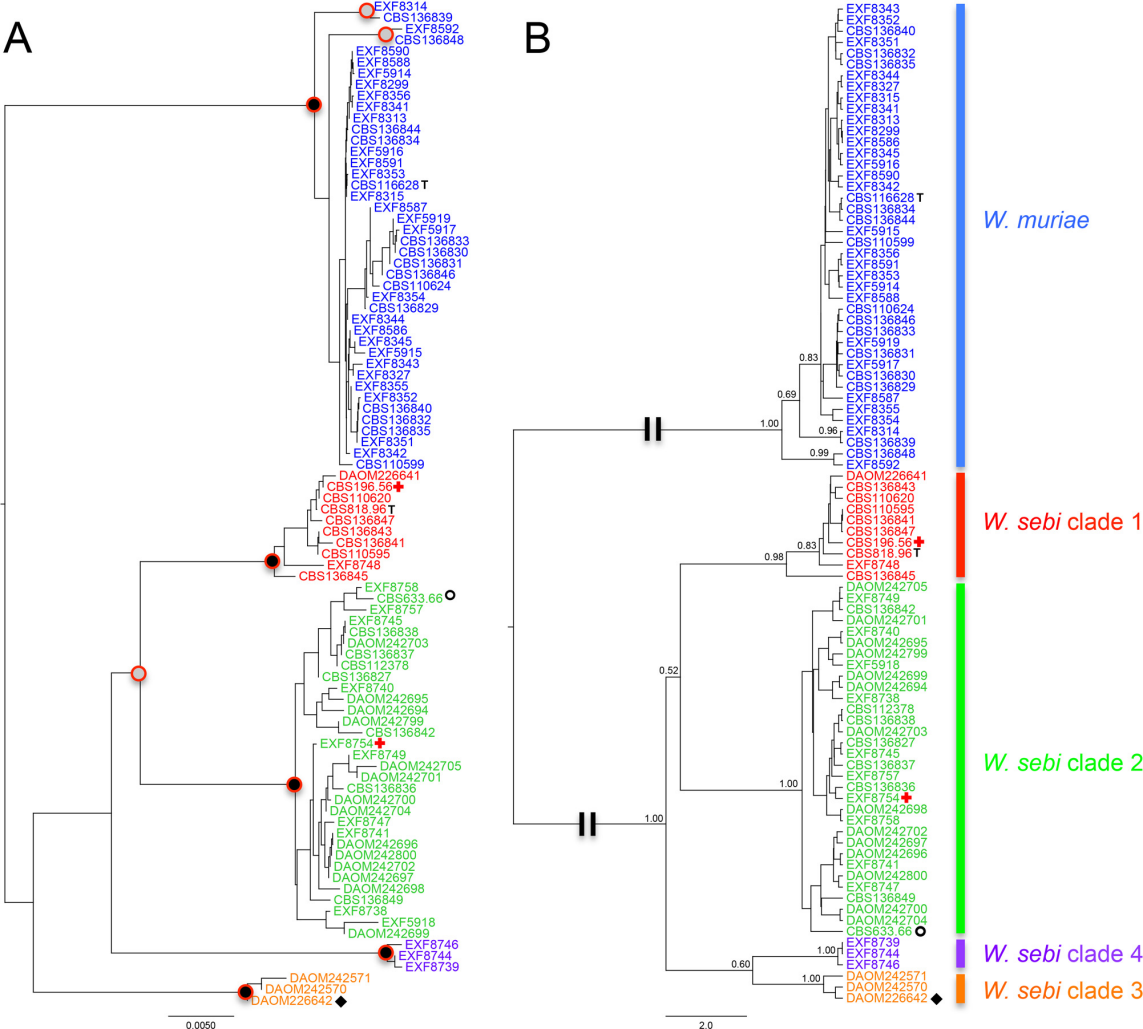
Multi-gene phylogenies. A. Neighbour joining tree based on the concatenated alignment. Nodes marked with black dots indicate the concordant groups detected consistently in all 5 genealogies. The grey dots indicate somewhat concordant groups detected 2 out of 5 genealogies. B. The inferred species tree from *BEAST. Posterior probabilities on important backbone nodes and strongly supported nodes (>0.80) are shown on the tree. Long branches were represented by a double break in the line. T = neotype strain, **○** = genome sequenced strain, ✚ = strains reported to cause subcutaneous lesions, ◆ = strain reported to produce metabolites that react to human antibodies.

We then performed a *BEAST analysis, which combines the information from multiple loci to yield a species tree. The nodes with strong support indicate the location of genealogical concordance, in essence the species limit. We considered nodes strongly supported if they received posterior probabilities (PP) ≥0.95. The *BEAST analysis strongly supported *W*. *sebi* clades 1, 2, 3, and 4 with *W*. *muriae* as a distinct clade. However, the branch length between *W*. *muriae* and all four *W*. *sebi* clades was long. Our species hypothesis was supported by the *BEAST analysis, but low posterior probability values were found in the backbone, which represent the confidence that can be applied to the relationships among the four *W*. *sebi* clades. This was consistent with results from the single gene phylogenies because backbone topologies varied from one gene to the next. The initial NJ tree marked with concordant nodes found across all single gene phylogenies and the *BEAST tree are summarized in Fig. 3. Supporting these results, the species delimitation analyses in BPP3 consistently reported a posterior probability of 0.99 to 1.00 for the five species (S2 File).

The clinically derived strains (CBS 196.56, EXF-8754, DAOM 226642) were in different clades, and there was no discernable support for a pathogenic clade in the WSSC.

### Geography and haplotype analysis

**Fig 4:**
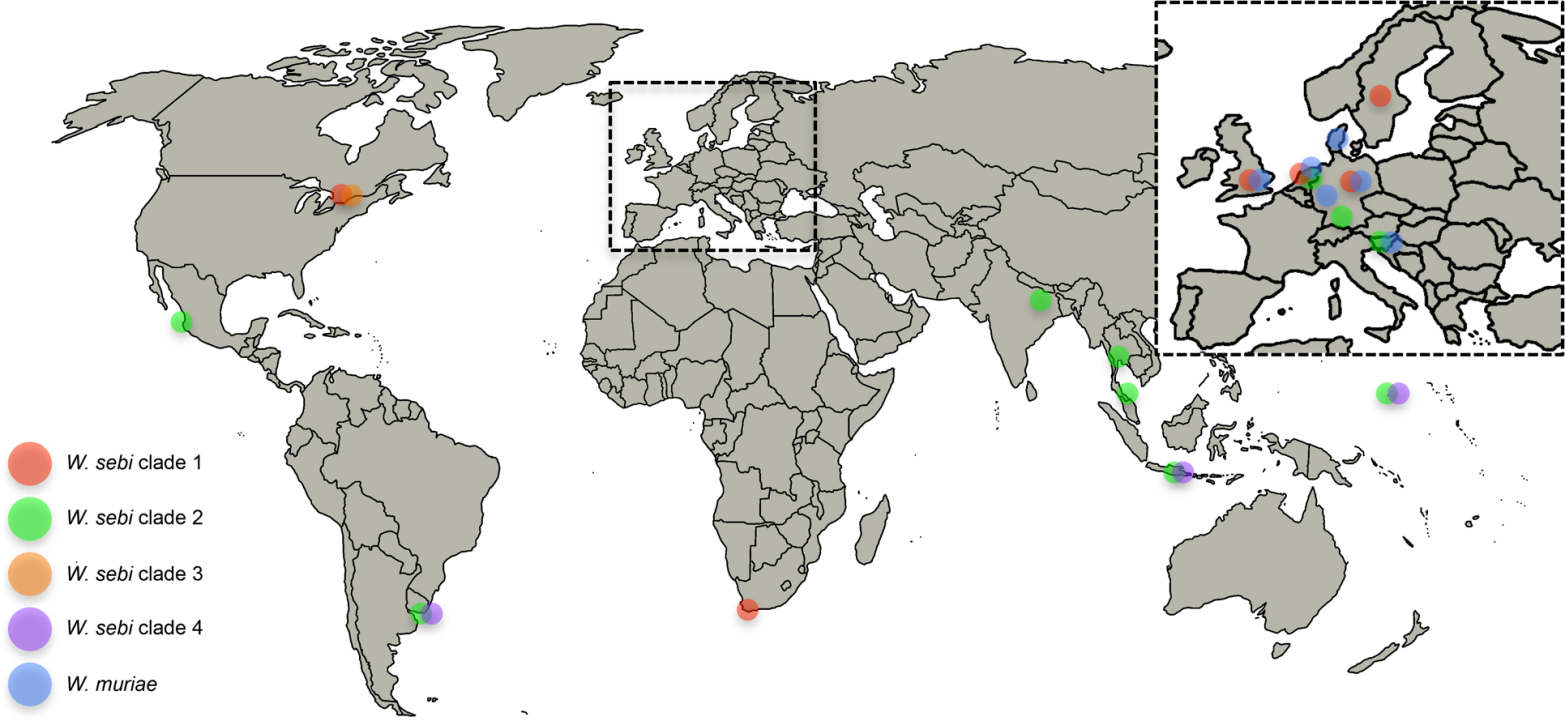
Approximate geographic location of our *Wallemia* isolates on the global map. Since most of our sampling came from Europe, the map of Europe is enlarged on the upper right hand corner.

Often, a single fungal species with an assumed cosmopolitan distribution is shown to be composed of multiple cryptic species that are geographically separated [14]. We mapped the approximate geographical origin of our strains, but there was no obvious geographical correlation with clade number (Fig. 4) among our samples.

To analyze the diversity of sampling, we used a haplotyping approach where each unique sequence (rather than each strain) was grouped. These results are summarized in S3 Table. The concatenated alignment used for haplotyping had 1255 sites, of which 176 were variable. Thirty-one distinct haplotypes were detected. Out these, 19 were singletons. Overall, the strains that grouped together as a clade in our phylogenetic analyses also grouped together in our haplotype analysis, but the different clades we postulated in our species hypothesis were further dissected. This is expected because a species should have different haplotypes. For example, *W*. *sebi* clade 1 included five haplotypes, *W*. *sebi* clade 2 was separated into 18 haplotypes and *W*. *muriae* had six distinct haplotypes. However, *W*. *sebi* clade 3 and clade 4 contained single haplotypes, which is an indication that these clades were not well sampled.

When we took into consideration the geography of the non-singleton haplotypes, we observed some patterns. Haplotype 1 and 4 are strictly European, haplotype 12 was found only in Micronesia while haplotype 17 and 18 contained a mixture of strains from Indonesia and Thailand, suggestive of a south Asian population. Haplotype 24, also known as *W*. *sebi* clade 3, included only Canadian strains. However, after adding singleton haplotypes and comparing all haplotypes, geographical ranges overlapped. Haplotype 13 contained a mixture of European and Micronesian strains. Haplotype 16 was also a mixture of strains from India, Indonesia and one strain from the Netherlands. Haplotype 25 corresponded to *W*. *sebi* clade 4 and it contained several strains from Uruguay, Micronesia and Indonesia. All of our strains identified as *W*. *muriae* came from Europe, although the species exhibited three different haplotypes.

## Discussion

Previous studies suggest that *Wallemia* is a common ubiquitous genus in the indoor environment, with *W*. *sebi* and *W*. *muriae* as the dominant species [3], [27], [38], [40], [41]. As shown by our investigation, *W*. *sebi* and *W*. *muriae* are the two most common species found indoors, confirming previous findings. We did not detect any novel species distantly related to *W*. *sebi* and *W*. *muriae sensu* Zalar et al. (2005) [3].

There is unexplained ITS diversity in *W*. *sebi* that hints at the existence of cryptic species, as suggested previously [3]. We were able to amplify the ITS locus in all species but it often failed to sequence. Fungi can have multiple copies of the ITS in tandem or even located on different chromosomes [70]. Because this region is not translated, multiple copies of ITS can evolve differently. However, concerted evolution may reduce infragenomic variation among copies, although some variation still exists [71], [72]. Lindner and Banik [73] showed that cloned ITS sequences of the same *Laetiporus* species (Polyporales, Basidiomycota) contained variation that could be interpreted as different species in a phylogenetic analysis. We speculate that *W*. *sebi* and *W*. *muriae* have multiple copies of the ITS region with high infragenomic variation. This could explain our inability to sequence the ITS marker with a high success rate.

We designed primers to amplify four other DNA markers (*MCM*7, *RPB*1, *RPB*2, and *TSR*1) and then conducted GCPSR-based multilocus phylogenetic analyses to detect cryptic species. We first conducted a neighbor joining phylogenetic analysis from a concatenated alignment to formulate our species hypothesis. Then, we tested genealogical concordance by reconstructing single gene phylogenies using four different methodologies and finally a multi-gene phylogenetic analysis using *BEAST. *Wallemia muriae* cohered as a monophyletic group in all analyses and was found only in Europe. However, this species may be in the early stages of speciation. Two basal clades to the main *W*. *muriae* group were detected in 2 of 5 genealogies (Fig. 3A). One of the clades consisted of strains EXF 8314 and CBS 136839 while the other included EXF 8592 and CBS 136848. Based on our data and given that only two strains comprised each clade, we could not see any supporting characters to suggest recognizing these clades as species at this time. However, four clades of *W*. *sebi* emerged from the phylogenetic analyses and fulfilled the requirements for phylogenetic species recognition. These clades are genealogically concordant and we found no disagreement. However, the ITS phylogeny did not support *W*. *sebi* clade 2 as a monophyletic group in any of the phylogenetic reconstructions. The high infragenomic variation allows for a high number of substitutions in a given site in short molecular time, possibly masking the phylogenetic signal, and could explain why *W*. *sebi* clade 2 was not monophyletic in the ITS phylogeny.

The phylogenetic signal produced from the other four markers (*MCM*7, *RPB*1, *RPB*2, and *TSR*1) is probably more accurate at representing genealogical concordance. Because all four DNA markers showed four clades of *W*. *sebi*, we suggest they should be recognized as different phylogenetic species. This was supported by our species delimitation analyses with BPP3. Although we used multiple loci to derive our phylogenetic species concept, only one of the four other protein coding markers is necessary to identify a *W*. *sebi* isolate to clade. Sequencing one other marker in addition to the official fungal barcode ITS would be more practical and economical. The sequence variation between species should exceed the variation within species. In DNA barcoding terms, this is referred to as the barcode gap. Of the four protein coding markers we tested, we recommend *TSR*1 as a secondary marker because of its clear barcode gap (Fig. 1).

We performed a haplotype analysis to estimate the haplotype diversity within the phylogenetic species. Generally, we did not find a strong link between geography and haplotype (S3 Table), but a pattern may emerge if more strains are studied. However, we observed that *W*. *muriae* was found strictly in Europe, *W*. *sebi* clade 3 was found in regions with temperate climates (Canada [33], S. Frasz and D. Miller pers. comm) and Finland [4]), and *W*. *sebi* clade 4 was detected in the subtropical countries (Uruguay, Micronesia, and Indonesia). However, *W*. *sebi* clade 1 and 2 seem to be distributed worldwide. Because of their overlapping ranges, there does not seem to be an indication of allopatric speciation, so these *Wallemia* species likely arose sympatrically or parapatrically from an ancestor population. The overlap in ranges suggests speciation may have occurred following colonization of new niches.

*Wallemia sebi* was suspected to cause allergies [28], [33] and perhaps subcutaneous lesions [25]. We did not find any evidence of a pathogenic species or haplotype of *Wallemia*. *Wallemia sebi* DAOM 226642 produces metabolites that react to human antibodies, whereas DAOM 242570 and DAOM 242571 lack compounds that bind to human antibodies [33]; all grouped phylogenetically in *W*. *sebi* clade 3. *Wallemia* is rarely reported as a pathogen and there are too few strains available to reveal any pattern. Its involvement in allergy is still enigmatic and requires further study.

*Wallemia* species lack striking morphological differences. They are defined by their physiology, especially their abilities to tolerate and grow on ranges of water activities. We describe and provide formal species names for the clades within the WSSC in a companion study [4], confirming the existence of the four phylogenetic species using strains from a broader range of habitats.

## Supporting Information

S1 Table Strain information and GenBank accession numbers. Sequences for *MCM*7, *TSR*1, *RPB*1 and *RPB*2 from strain CBS 633.66 were extracted from the Joint Genome Institute (JGI) MycoCosm site.
(XLSX)

S2 Table Primer sequences. (XLSX)

S3 Table List of haplotypes. (XLSX)

S4 Table Support values for monophyly of each clade in our species hypothesis. Low or unsupported values are highlighted in yellow.
(XLSX)

S1 File All trees resulting from single gene phylogenetic analyses. Only support values of greater than 70% or 0.70 are shown.
(PDF)

S2 File BPP3 analyses. (ZIP)
