# Supplementary material for: Application of the Phylogenetic Species Concept to Wallemia sebi from House Dust and Indoor Air Revealed by Multi-Locus Genealogical Concordance
Source: PLoS One. 2015 Mar 23;10(3):e0120894. doi: 10.1371/journal.pone.0120894 (PMC4370657; doi:10.1371/journal.pone.0120894)
Supplement: S1 File — Only support values of greater than 70% or 0.70 are shown. (PDF) [file pone.0120894.s005.pdf]

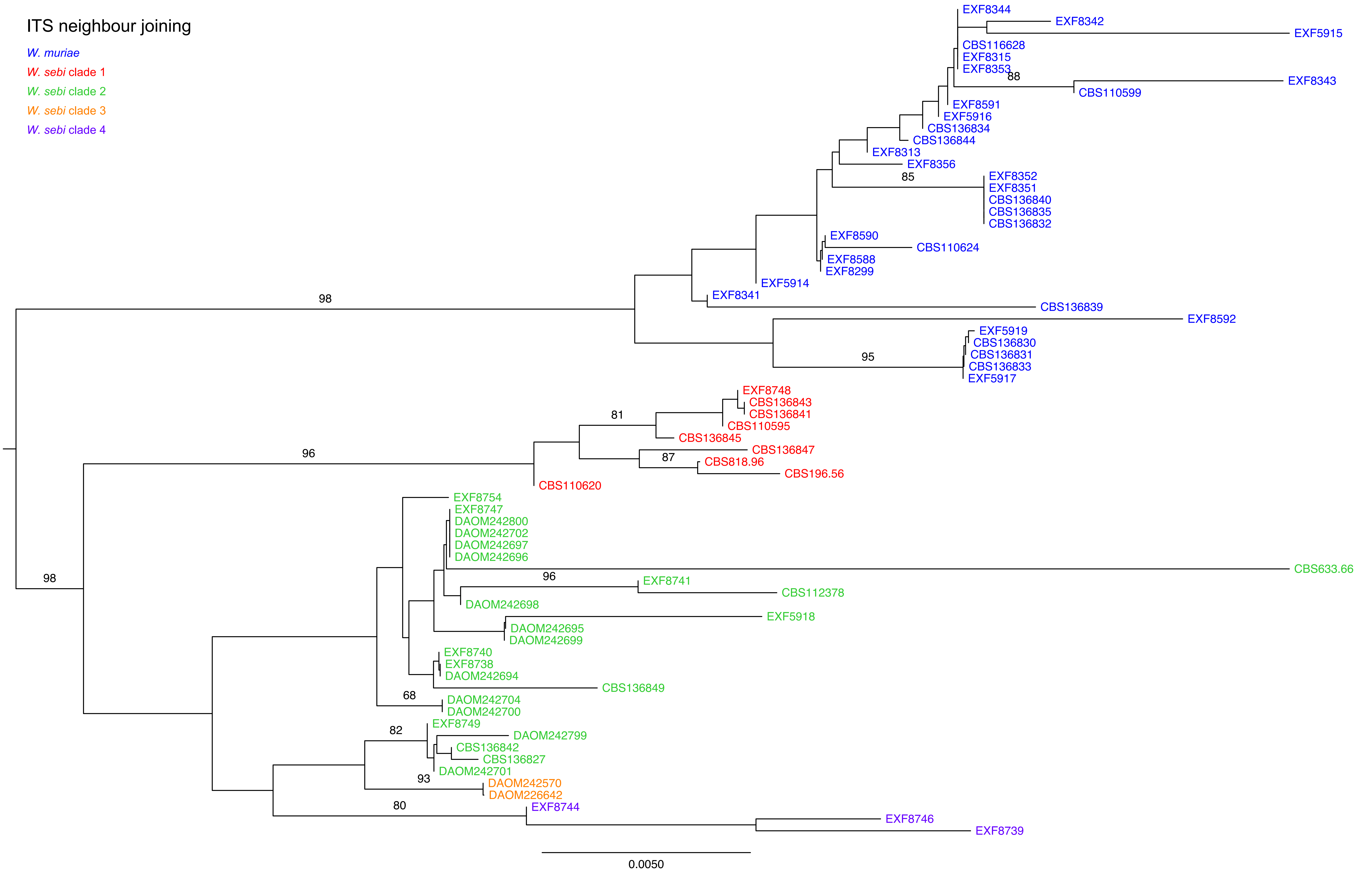
ITS neighbour joining
W. muriae
W. sebi clade 1
W. sebi clade 2
W. sebi clade 3
W. sebi clade 4
EXF8344
EXF8342
EXF5915
CBS116628
EXF8315
EXF8353
88
EXF8343
CBS110599
EXF8591
EXF5916
CBS136834
CBS136844
EXF8313
EXF8356
85
EXF8352
EXF8351
CBS136840
CBS136835
CBS136832
EXF8590
CBS110624
EXF8588
EXF8299
EXF5914
EXF8341
CBS136839
98
EXF8592
EXF5919
CBS136830
CBS136831
95
CBS136833
EXF5917
EXF8748
CBS136843
CBS136841
81
CBS110595
CBS136845
96
CBS136847
87
CBS818.96
CBS196.56
CBS110620
EXF8754
EXF8747
DAOM242800
DAOM242702
DAOM242697
DAOM242696
CBS633.66
98
96
EXF8741
CBS112378
DAOM242698
EXF5918
DAOM242695
DAOM242699
EXF8740
EXF8738
DAOM242694
CBS136849
68
DAOM242704
DAOM242700
EXF8749
82
DAOM242799
CBS136842
CBS136827
DAOM242701
93
DAOM242570
DAOM226642
80
EXF8744
EXF8746
EXF8739
0.0050

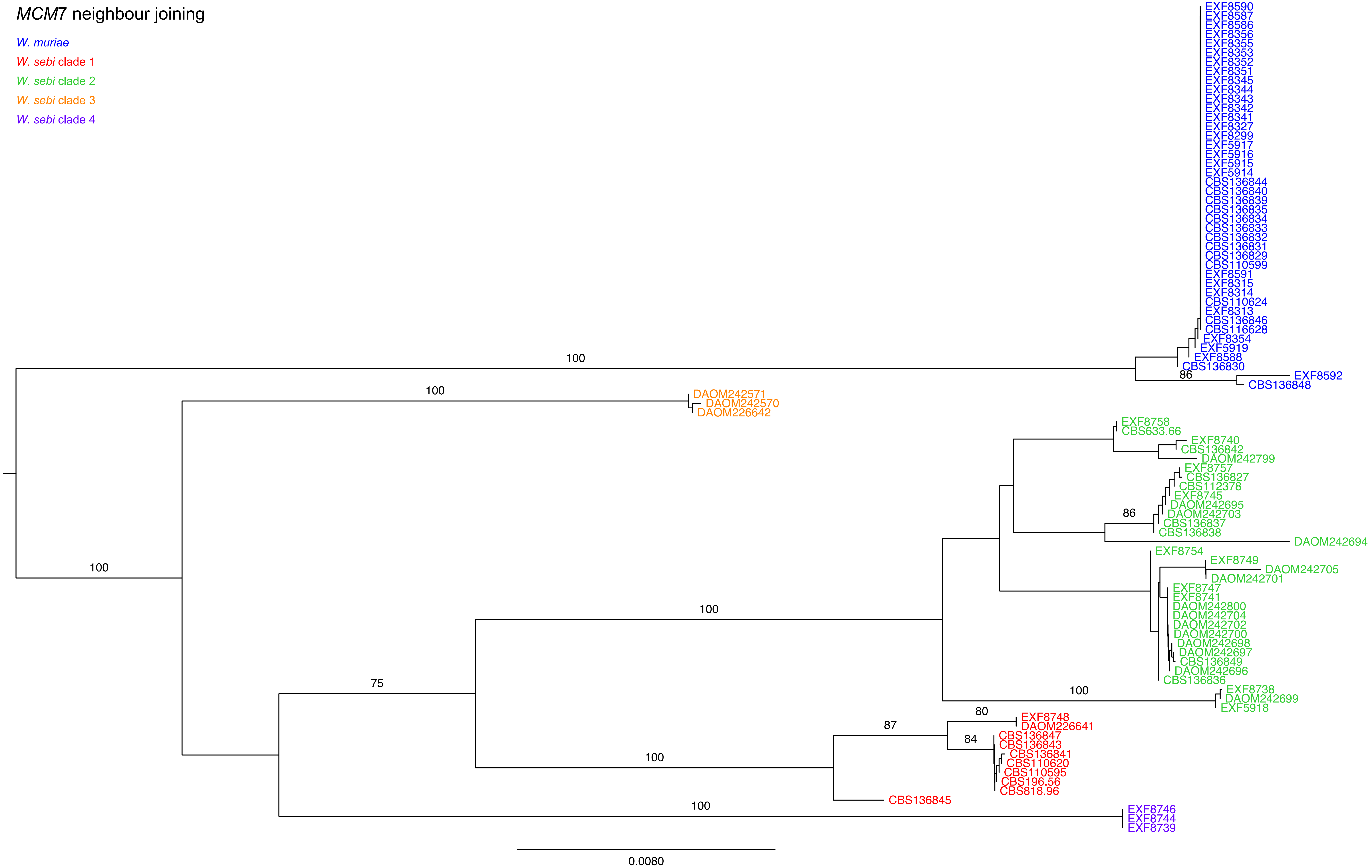

MCM7 neighbour joining
W. muriae
W. sebi clade 1
W. sebi clade 2
W. sebi clade 3
W. sebi clade 4
EXF8590
EXF8587
EXF8586
EXF8356
EXF8355
EXF8353
EXF8352
EXF8351
EXF8345
EXF8344
EXF8343
EXF8342
EXF8341
EXF8327
EXF8299
EXF5917
EXF5916
EXF5915
EXF5914
CBS136844
CBS136840
CBS136839
CBS136835
CBS136834
CBS136833
CBS136832
CBS136831
CBS136829
CBS110599
EXF8591
EXF8315
EXF8314
CBS110624
EXF8313
CBS136846
CBS116628
EXF8354
EXF5919
EXF8588
CBS136830
EXF8592
CBS136848
100
86
DAOM242571
DAOM242570
DAOM226642
100
EXF8758
CBS633.66
EXF8740
CBS136842
DAOM242799
EXF8757
CBS136827
CBS112378
EXF8745
DAOM242695
DAOM242703
CBS136837
CBS136838
86
DAOM242694
EXF8754
EXF8749
DAOM242705
DAOM242701
EXF8747
EXF8741
DAOM242800
DAOM242704
DAOM242702
DAOM242700
DAOM242698
DAOM242697
CBS136849
DAOM242696
CBS136836
100
EXF8738
DAOM242699
EXF5918
100
100
75
80
EXF8748
DAOM226641
87
84
CBS136847
CBS136843
CBS136841
CBS110620
CBS110595
CBS196.56
CBS818.96
100
CBS136845
100
EXF8746
EXF8744
EXF8739
0.0080

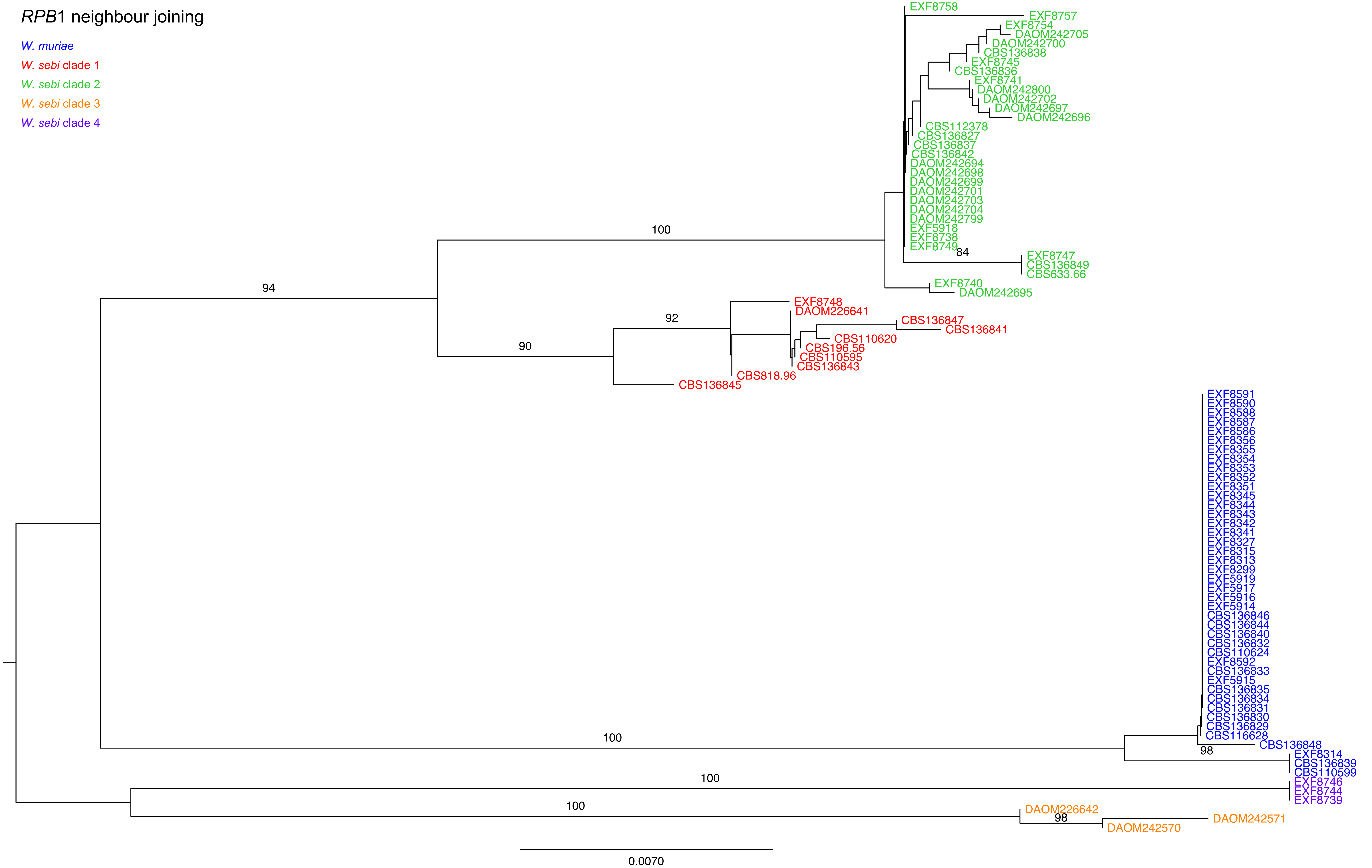

RPB1 neighbour joining
W. muriae
W. sebi clade 1
W. sebi clade 2
W. sebi clade 3
W. sebi clade 4
EXF8758
EXF8757
EXF8754
DAOM242705
DAOM242700
CBS136838
EXF8745
CBS136836
EXF8741
DAOM242800
DAOM242702
DAOM242697
DAOM242696
CBS112378
CBS136827
CBS136837
CBS136842
DAOM242694
DAOM242698
DAOM242699
DAOM242701
DAOM242703
DAOM242704
DAOM242799
EXF5918
EXF8738
EXF8749
84
EXF8747
CBS136849
CBS633.66
EXF8740
DAOM242695
100
94
EXF8748
DAOM226641
CBS136847
CBS136841
CBS110620
CBS196.56
CBS110595
CBS136843
CBS818.96
CBS136845
92
90
EXF8591
EXF8590
EXF8588
EXF8587
EXF8586
EXF8356
EXF8355
EXF8354
EXF8353
EXF8352
EXF8351
EXF8345
EXF8344
EXF8343
EXF8342
EXF8341
EXF8327
EXF8315
EXF8313
EXF8299
EXF5919
EXF5917
EXF5916
EXF5914
CBS136846
CBS136844
CBS136840
CBS136832
CBS110624
EXF8592
CBS136833
EXF5915
CBS136835
CBS136834
CBS136831
CBS136830
CBS136829
CBS116628
CBS136848
98
EXF8314
CBS136839
CBS110599
100
EXF8746
EXF8744
EXF8739
100
DAOM226642
98
DAOM242571
DAOM242570
100
0.0070

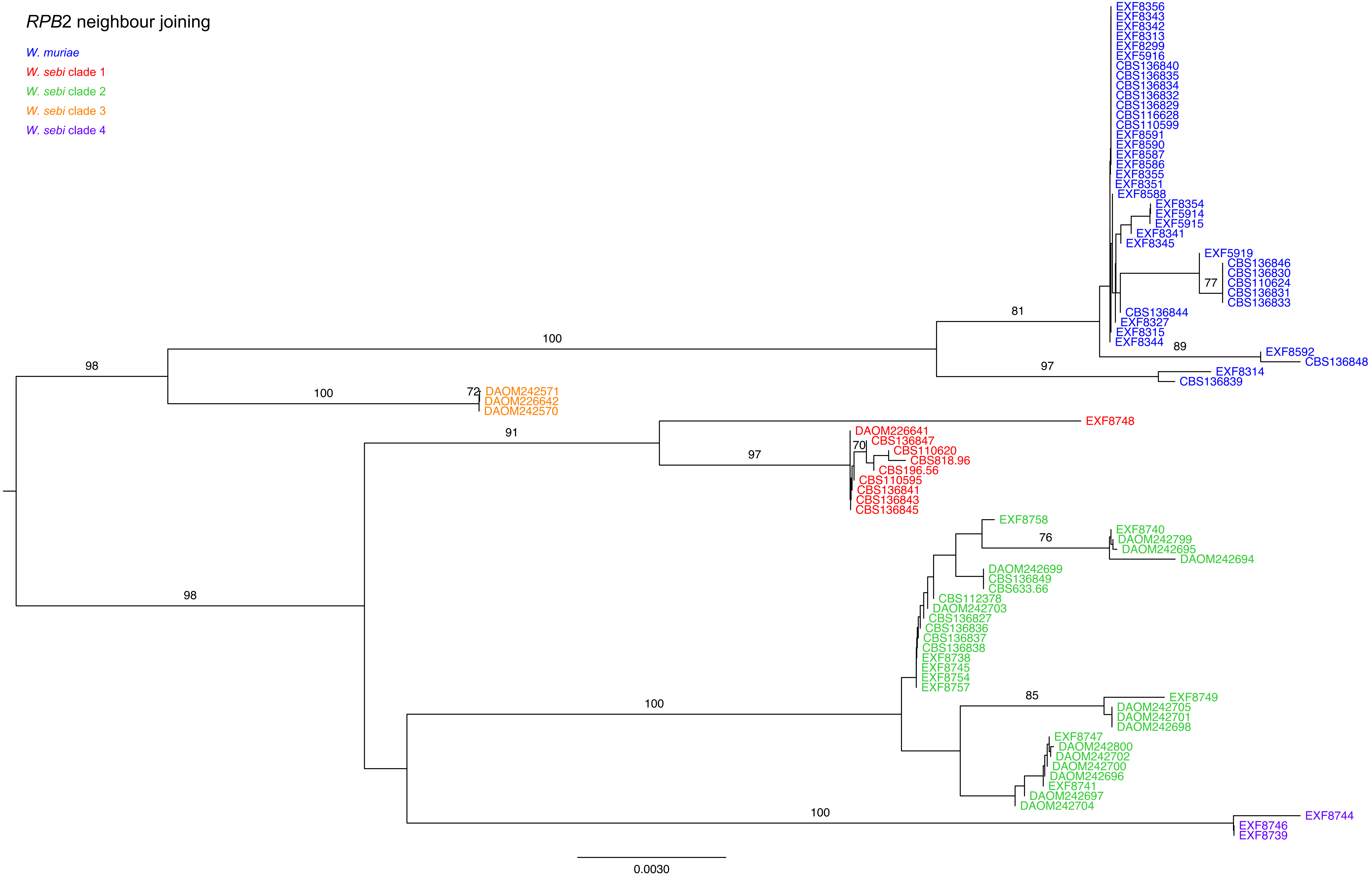
RPB2 neighbour joining
W. muriae
W. sebi clade 1
W. sebi clade 2
W. sebi clade 3
W. sebi clade 4
EXF8356
EXF8343
EXF8342
EXF8313
EXF8299
EXF5916
CBS136840
CBS136835
CBS136834
CBS136832
CBS136829
CBS116628
CBS110599
EXF8591
EXF8590
EXF8587
EXF8586
EXF8355
EXF8351
EXF8588
EXF8354
EXF5914
EXF5915
EXF8341
EXF8345
EXF5919
CBS136846
CBS136830
CBS110624
CBS136831
CBS136833
77
CBS136844
EXF8327
EXF8315
EXF8344
89
81
100
98
EXF8592
CBS136848
97
EXF8314
CBS136839
100
72
DAOM242571
DAOM226642
DAOM242570
EXF8748
91
97
DAOM226641
CBS136847
70
CBS110620
CBS818.96
CBS196.56
CBS110595
CBS136841
CBS136843
CBS136845
EXF8758
EXF8740
DAOM242799
76
DAOM242695
DAOM242694
DAOM242699
CBS136849
CBS633.66
CBS112378
DAOM242703
CBS136827
CBS136836
CBS136837
CBS136838
EXF8738
EXF8745
EXF8754
EXF8757
98
100
85
EXF8749
DAOM242705
DAOM242701
DAOM242698
EXF8747
DAOM242800
DAOM242702
DAOM242700
DAOM242696
EXF8741
DAOM242697
DAOM242704
100
EXF8744
EXF8746
EXF8739
0.0030

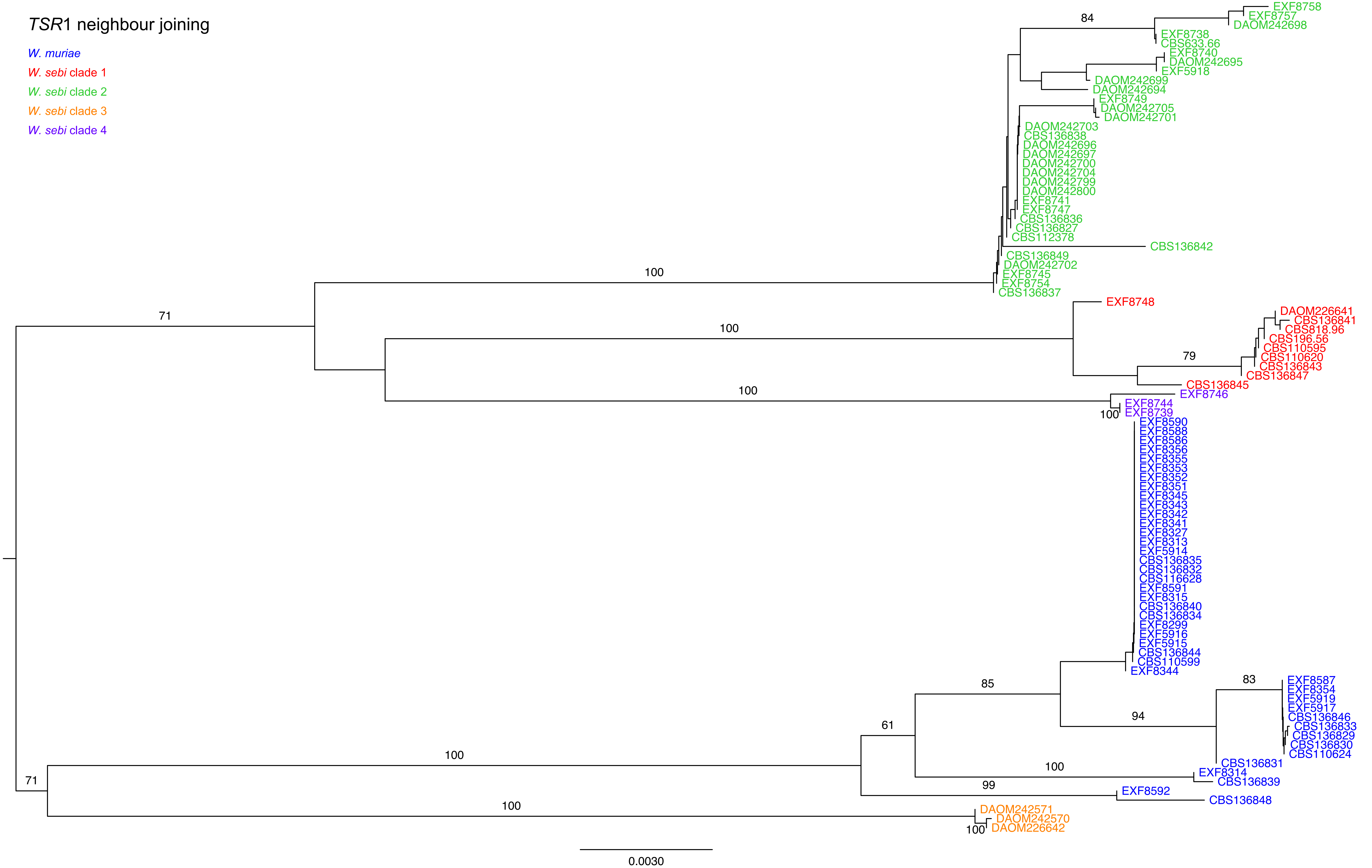
TSR1 neighbour joining
W. muriae
W. sebi clade 1
W. sebi clade 2
W. sebi clade 3
W. sebi clade 4
EXF8758
EXF8757
DAOM242698
EXF8738
CBS633.66
EXF8740
DAOM242695
EXF5918
DAOM242699
DAOM242694
EXF8749
DAOM242705
DAOM242701
DAOM242703
CBS136838
DAOM242696
DAOM242697
DAOM242700
DAOM242704
DAOM242799
DAOM242800
EXF8741
EXF8747
CBS136836
CBS136827
CBS112378
CBS136842
CBS136849
DAOM242702
EXF8745
EXF8754
CBS136837
EXF8748
DAOM226641
CBS136841
CBS818.96
CBS196.56
CBS110595
CBS110620
CBS136843
CBS136847
CBS136845
EXF8746
EXF8744
EXF8739
EXF8590
EXF8588
EXF8586
EXF8356
EXF8355
EXF8353
EXF8352
EXF8351
EXF8345
EXF8343
EXF8342
EXF8341
EXF8327
EXF8313
EXF5914
CBS136835
CBS136832
CBS116628
EXF8591
EXF8315
CBS136840
CBS136834
EXF8299
EXF5916
EXF5915
CBS136844
CBS110599
EXF8344
EXF8587
EXF8354
EXF5919
EXF5917
CBS136846
CBS136833
CBS136829
CBS136830
CBS110624
CBS136831
EXF8314
CBS136839
EXF8592
CBS136848
DAOM242571
DAOM242570
DAOM226642
84
100
100
79
100
100
71
85
94
83
61
100
100
99
71
100
100
0.0030

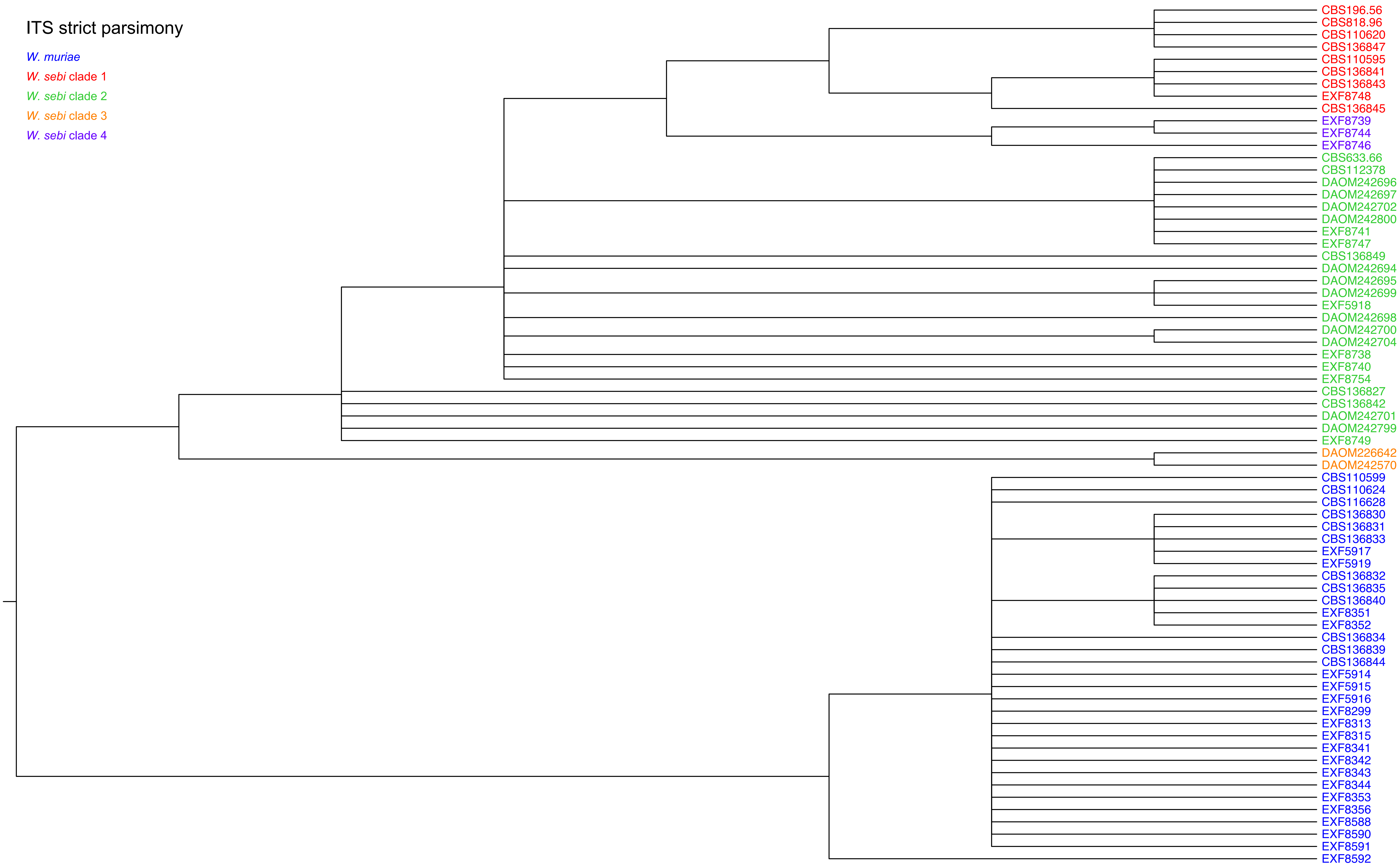

ITS strict parsimony
W. muriae
W. sebi clade 1
W. sebi clade 2
W. sebi clade 3
W. sebi clade 4
CBS196.56
CBS818.96
CBS110620
CBS136847
CBS110595
CBS136841
CBS136843
EXF8748
CBS136845
EXF8739
EXF8744
EXF8746
CBS633.66
CBS112378
DAOM242696
DAOM242697
DAOM242702
DAOM242800
EXF8741
EXF8747
CBS136849
DAOM242694
DAOM242695
DAOM242699
EXF5918
DAOM242698
DAOM242700
DAOM242704
EXF8738
EXF8740
EXF8754
CBS136827
CBS136842
DAOM242701
DAOM242799
EXF8749
DAOM226642
DAOM242570
CBS110599
CBS110624
CBS116628
CBS136830
CBS136831
CBS136833
EXF5917
EXF5919
CBS136832
CBS136835
CBS136840
EXF8351
EXF8352
CBS136834
CBS136839
CBS136844
EXF5914
EXF5915
EXF5916
EXF8299
EXF8313
EXF8315
EXF8341
EXF8342
EXF8343
EXF8344
EXF8353
EXF8356
EXF8588
EXF8590
EXF8591
EXF8592

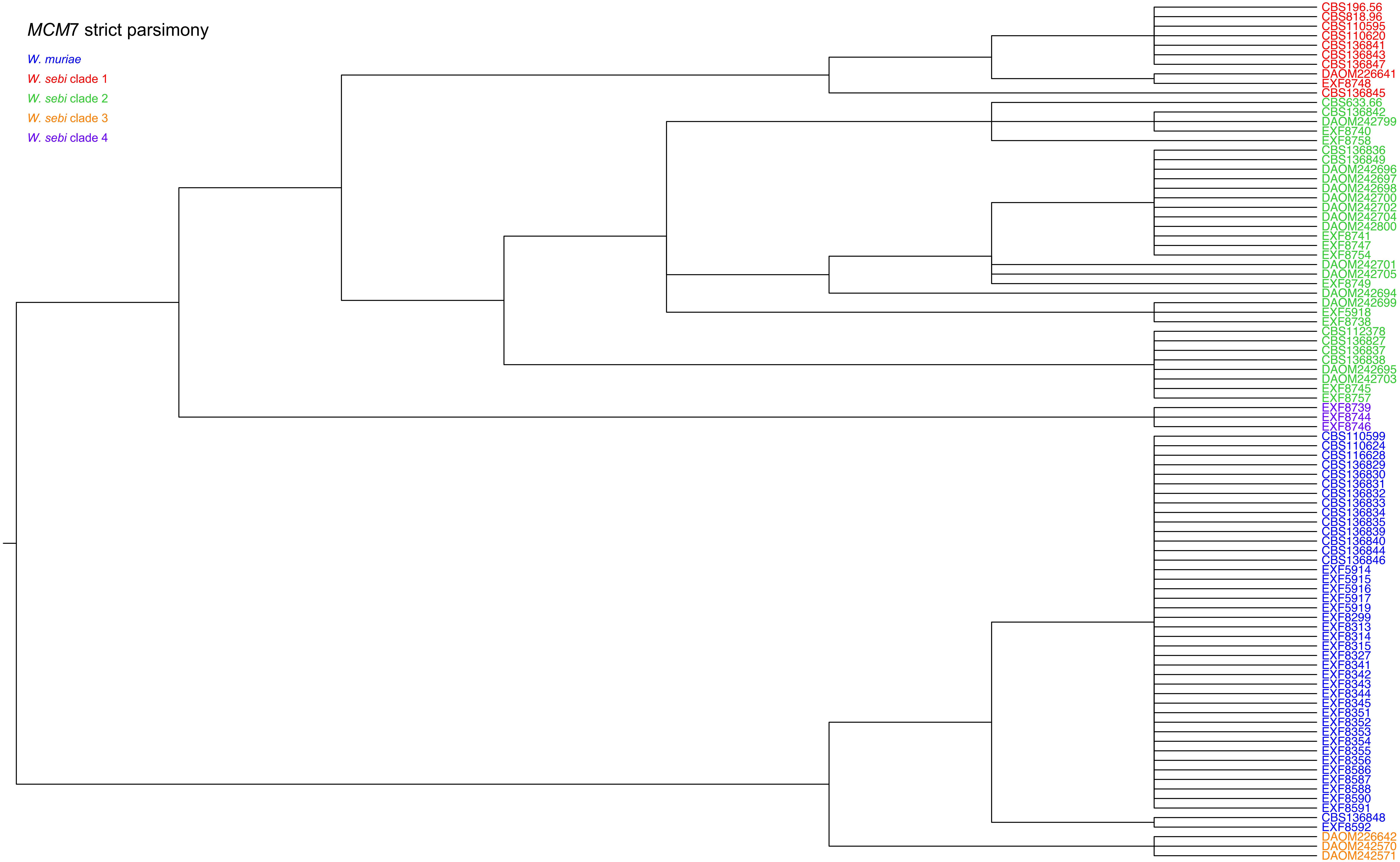

MCM7 strict parsimony
W. muriae
W. sebi clade 1
W. sebi clade 2
W. sebi clade 3
W. sebi clade 4
CBS196.56
CBS818.96
CBS110595
CBS110620
CBS136841
CBS136843
CBS136847
DAOM226641
EXF8748
CBS136845
CBS633.66
CBS136842
DAOM242799
EXF8740
EXF8758
CBS136836
CBS136849
DAOM242696
DAOM242697
DAOM242698
DAOM242700
DAOM242702
DAOM242704
DAOM242800
EXF8741
EXF8747
EXF8754
DAOM242701
DAOM242705
EXF8749
DAOM242694
DAOM242699
EXF5918
EXF8738
CBS112378
CBS136827
CBS136837
CBS136838
DAOM242695
DAOM242703
EXF8745
EXF8757
EXF8739
EXF8744
EXF8746
CBS110599
CBS110624
CBS116628
CBS136829
CBS136830
CBS136831
CBS136832
CBS136833
CBS136834
CBS136835
CBS136839
CBS136840
CBS136844
CBS136846
EXF5914
EXF5915
EXF5916
EXF5917
EXF5919
EXF8299
EXF8313
EXF8314
EXF8315
EXF8327
EXF8341
EXF8342
EXF8343
EXF8344
EXF8345
EXF8351
EXF8352
EXF8353
EXF8354
EXF8355
EXF8356
EXF8586
EXF8587
EXF8588
EXF8590
EXF8591
CBS136848
EXF8592
DAOM226642
DAOM242570
DAOM242571

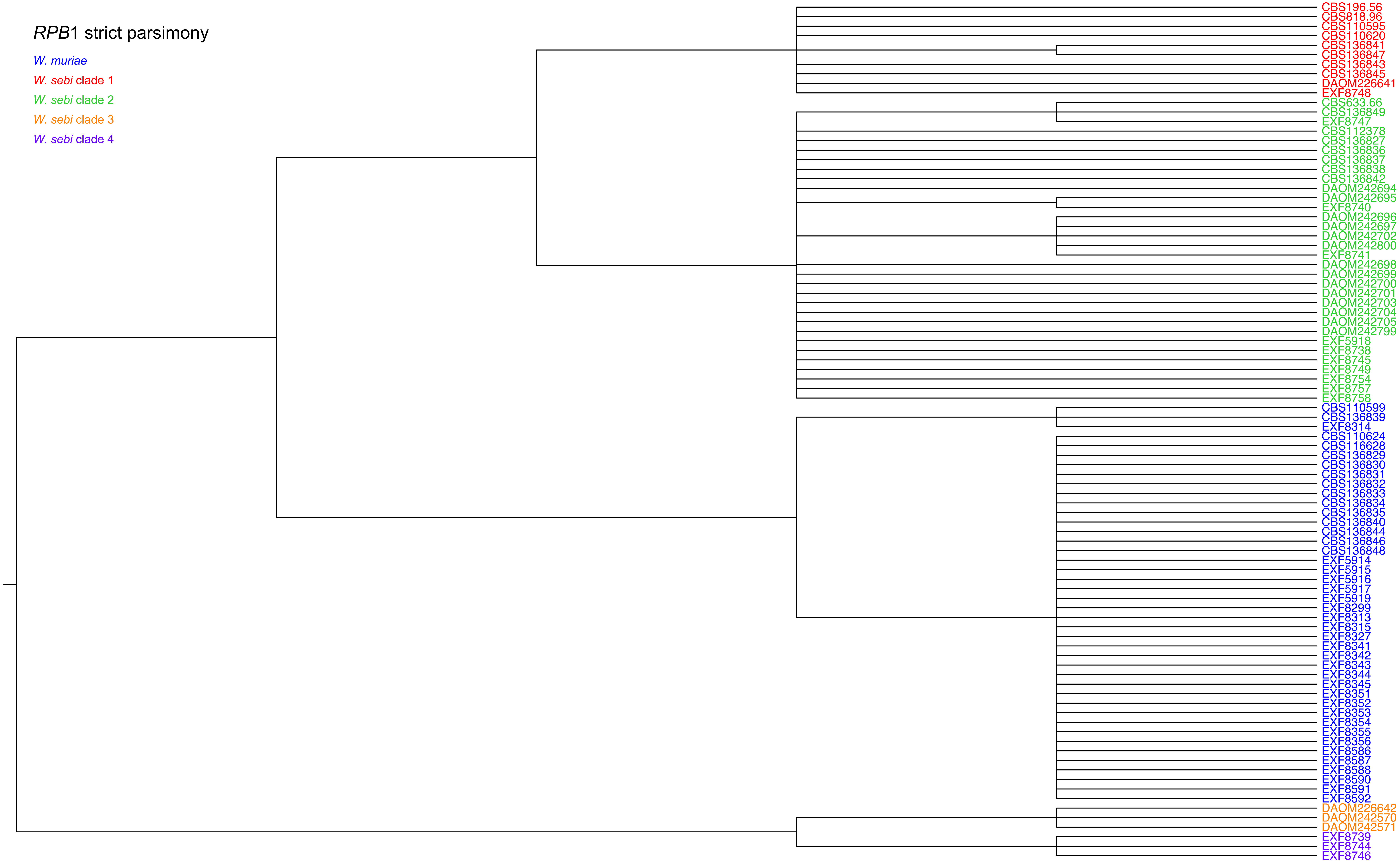
*RPB*1 strict parsimony
W. muriae
W. sebi clade 1
W. sebi clade 2
W. sebi clade 3
W. sebi clade 4
CBS196.56
CBS818.96
CBS110595
CBS110620
CBS136841
CBS136847
CBS136843
CBS136845
DAOM226641
EXF8748
CBS633.66
CBS136849
EXF8747
CBS112378
CBS136827
CBS136836
CBS136837
CBS136838
CBS136842
DAOM242694
DAOM242695
EXF8740
DAOM242696
DAOM242697
DAOM242702
DAOM242800
EXF8741
DAOM242698
DAOM242699
DAOM242700
DAOM242701
DAOM242703
DAOM242704
DAOM242705
DAOM242799
EXF5918
EXF8738
EXF8745
EXF8749
EXF8754
EXF8757
EXF8758
CBS110599
CBS136839
EXF8314
CBS110624
CBS116628
CBS136829
CBS136830
CBS136831
CBS136832
CBS136833
CBS136834
CBS136835
CBS136840
CBS136844
CBS136846
CBS136848
EXF5914
EXF5915
EXF5916
EXF5917
EXF5919
EXF8299
EXF8313
EXF8315
EXF8327
EXF8341
EXF8342
EXF8343
EXF8344
EXF8345
EXF8351
EXF8352
EXF8353
EXF8354
EXF8355
EXF8356
EXF8586
EXF8587
EXF8588
EXF8590
EXF8591
EXF8592
DAOM226642
DAOM242570
DAOM242571
EXF8739
EXF8744
EXF8746

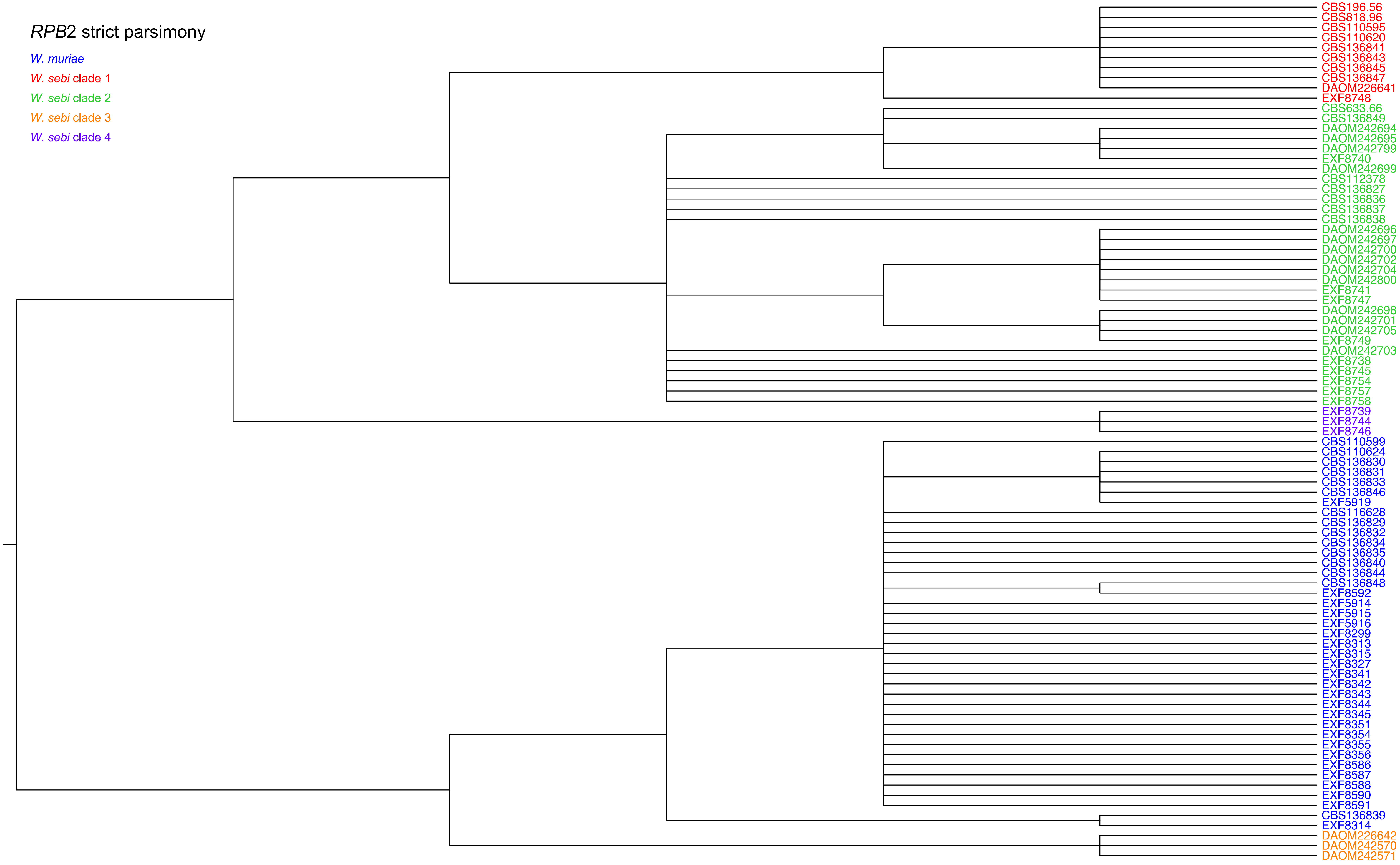
RPB2 strict parsimony
W. muriae
W. sebi clade 1
W. sebi clade 2
W. sebi clade 3
W. sebi clade 4
CBS196.56
CBS818.96
CBS110595
CBS110620
CBS136841
CBS136843
CBS136845
CBS136847
DAOM226641
EXF8748
CBS633.66
CBS136849
DAOM242694
DAOM242695
DAOM242799
EXF8740
DAOM242699
CBS112378
CBS136827
CBS136836
CBS136837
CBS136838
DAOM242696
DAOM242697
DAOM242700
DAOM242702
DAOM242704
DAOM242800
EXF8741
EXF8747
DAOM242698
DAOM242701
DAOM242705
EXF8749
DAOM242703
EXF8738
EXF8745
EXF8754
EXF8757
EXF8758
EXF8739
EXF8744
EXF8746
CBS110599
CBS110624
CBS136830
CBS136831
CBS136833
CBS136846
EXF5919
CBS116628
CBS136829
CBS136832
CBS136834
CBS136835
CBS136840
CBS136844
CBS136848
EXF8592
EXF5914
EXF5915
EXF5916
EXF8299
EXF8313
EXF8315
EXF8327
EXF8341
EXF8342
EXF8343
EXF8344
EXF8345
EXF8351
EXF8354
EXF8355
EXF8356
EXF8586
EXF8587
EXF8588
EXF8590
EXF8591
CBS136839
EXF8314
DAOM226642
DAOM242570
DAOM242571

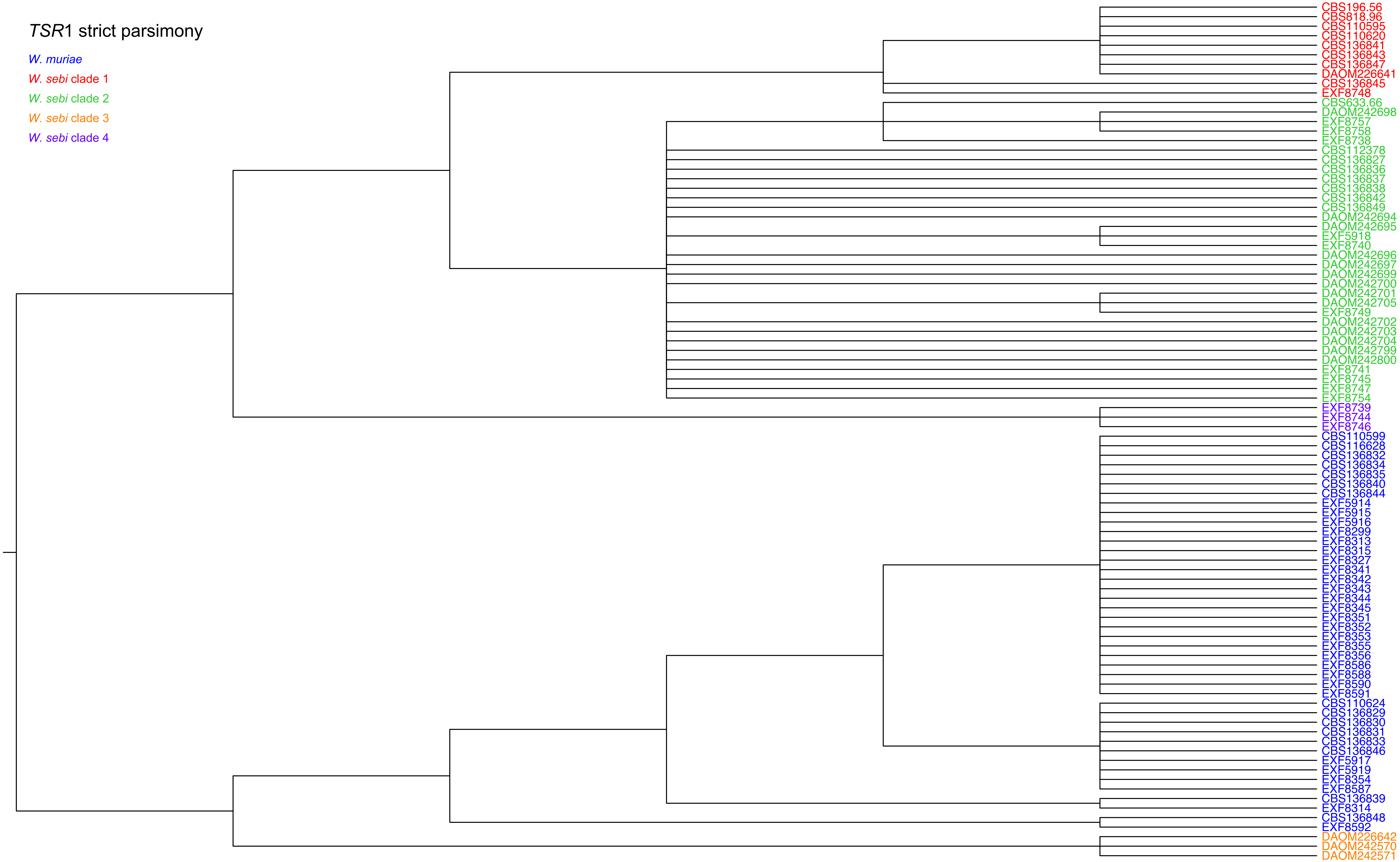
*TSR*1 strict parsimony
W. muriae
W. sebi clade 1
W. sebi clade 2
W. sebi clade 3
W. sebi clade 4
CBS196.56
CBS818.96
CBS110595
CBS110620
CBS136841
CBS136843
CBS136847
DAOM226641
CBS136845
EXF8748
CBS633.66
DAOM242698
EXF8757
EXF8758
EXF8738
CBS112378
CBS136827
CBS136836
CBS136837
CBS136838
CBS136842
CBS136849
DAOM242694
DAOM242695
EXF5918
EXF8740
DAOM242696
DAOM242697
DAOM242699
DAOM242700
DAOM242701
DAOM242705
EXF8749
DAOM242702
DAOM242703
DAOM242704
DAOM242799
DAOM242800
EXF8741
EXF8745
EXF8747
EXF8754
EXF8739
EXF8744
EXF8746
CBS110599
CBS116628
CBS136832
CBS136834
CBS136835
CBS136840
CBS136844
EXF5914
EXF5915
EXF5916
EXF8299
EXF8313
EXF8315
EXF8327
EXF8341
EXF8342
EXF8343
EXF8344
EXF8345
EXF8351
EXF8352
EXF8353
EXF8355
EXF8356
EXF8586
EXF8588
EXF8590
EXF8591
CBS110624
CBS136829
CBS136830
CBS136831
CBS136833
CBS136846
EXF5917
EXF5919
EXF8354
EXF8587
CBS136839
EXF8314
CBS136848
EXF8592
DAOM226642
DAOM242570
DAOM242571

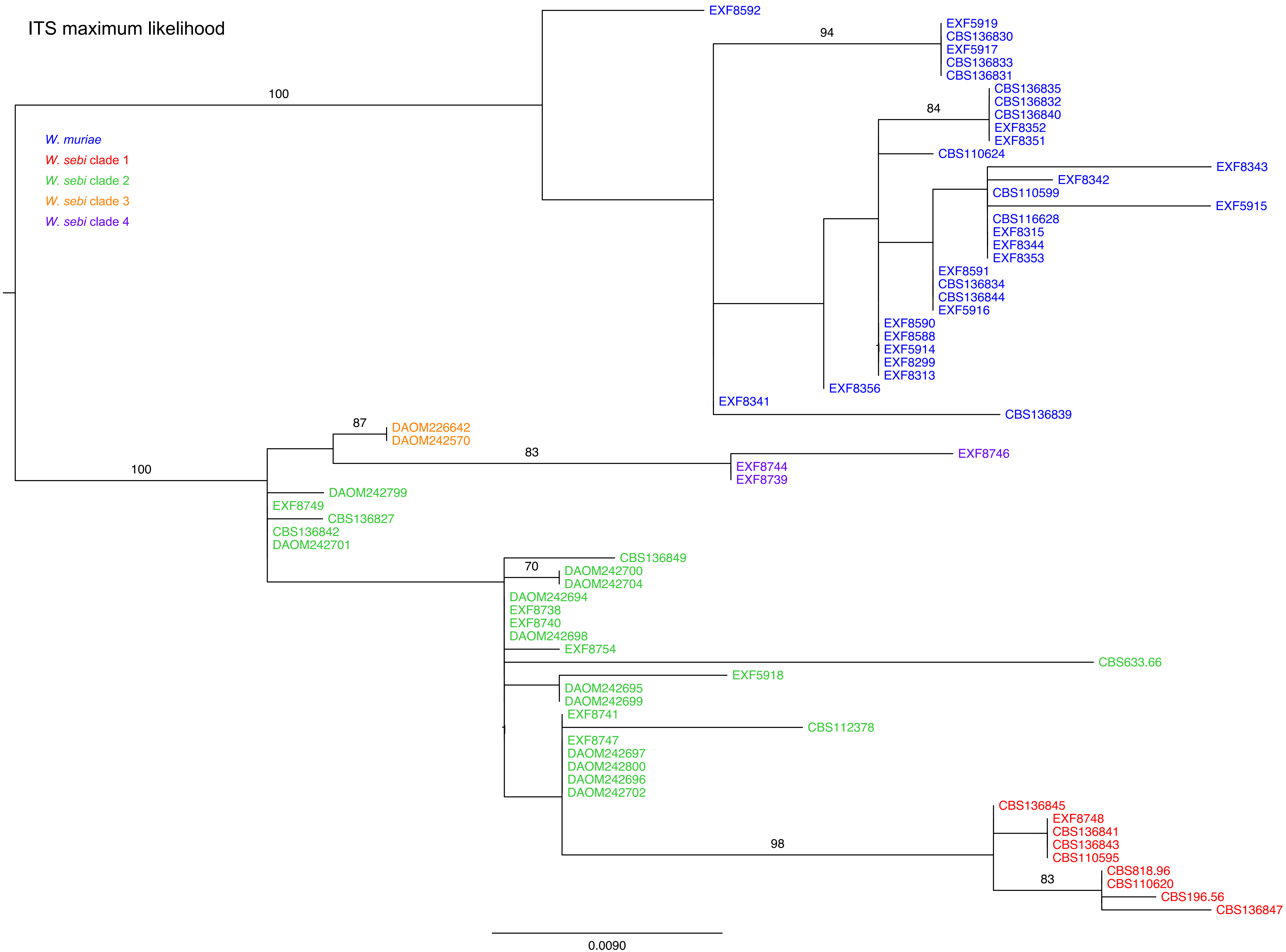

ITS maximum likelihood
W. muriae
W. sebi clade 1
W. sebi clade 2
W. sebi clade 3
W. sebi clade 4
100
EXF8592
94
EXF5919
CBS136830
EXF5917
CBS136833
CBS136831
84
CBS136835
CBS136832
CBS136840
EXF8352
EXF8351
CBS110624
EXF8343
EXF8342
CBS110599
EXF5915
CBS116628
EXF8315
EXF8344
EXF8353
EXF8591
CBS136834
CBS136844
EXF5916
EXF8590
EXF8588
EXF5914
EXF8299
EXF8313
EXF8356
EXF8341
CBS136839
87
DAOM226642
DAOM242570
83
EXF8746
EXF8744
EXF8739
100
DAOM242799
EXF8749
CBS136827
CBS136842
DAOM242701
CBS136849
70
DAOM242700
DAOM242704
DAOM242694
EXF8738
EXF8740
DAOM242698
EXF8754
CBS633.66
EXF5918
DAOM242695
DAOM242699
EXF8741
CBS112378
EXF8747
DAOM242697
DAOM242800
DAOM242696
DAOM242702
CBS136845
EXF8748
CBS136841
CBS136843
CBS110595
98
CBS818.96
CBS110620
83
CBS196.56
CBS136847
0.0090

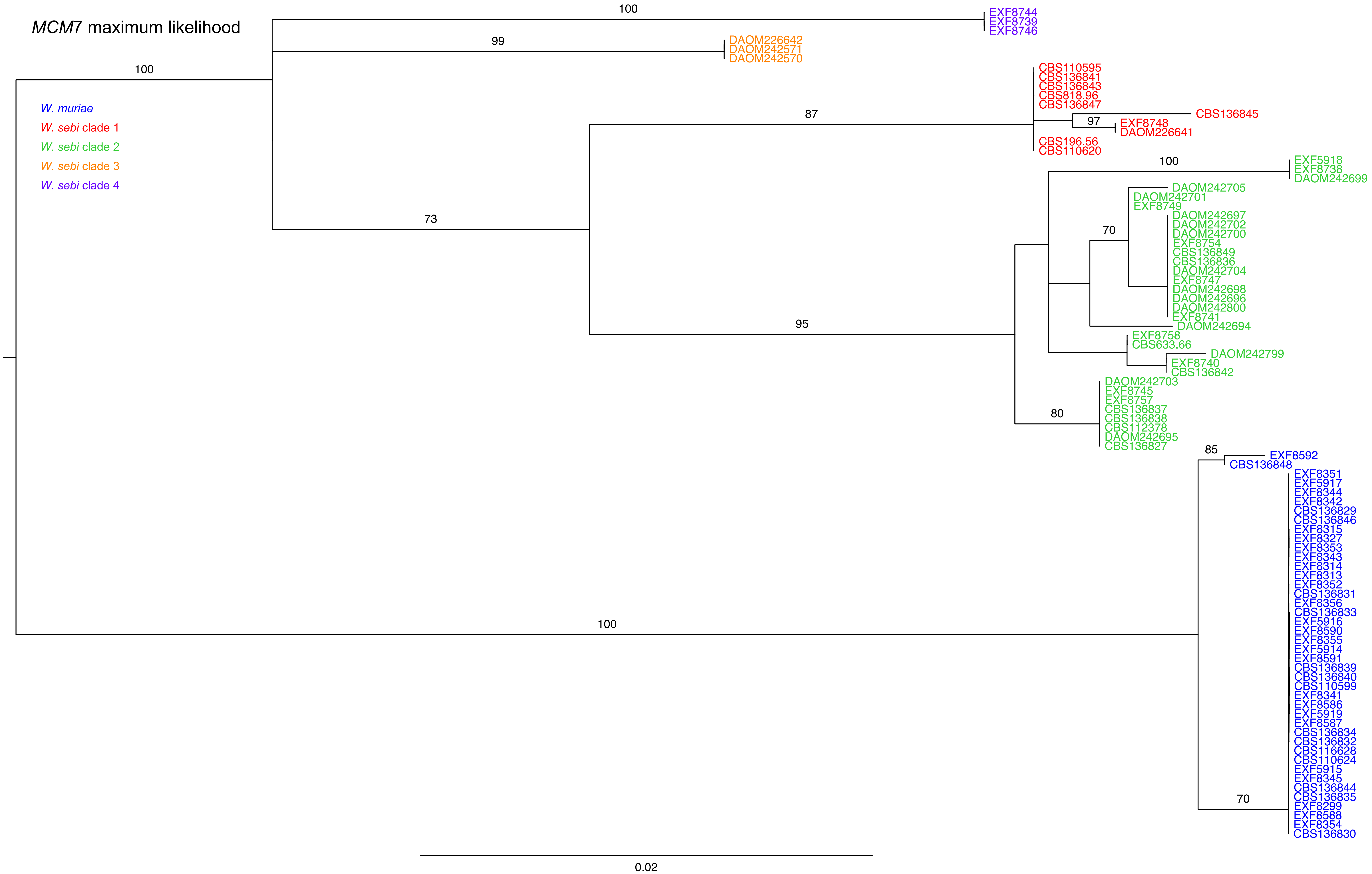

MCM7 maximum likelihood
W. muriae
W. sebi clade 1
W. sebi clade 2
W. sebi clade 3
W. sebi clade 4
100
100
99
87
97
100
70
73
95
80
85
100
70
0.02
EXF8744
EXF8739
EXF8746
DAOM226642
DAOM242571
DAOM242570
CBS110595
CBS136841
CBS136843
CBS818.96
CBS136847
CBS136845
EXF8748
DAOM226641
CBS196.56
CBS110620
EXF5918
EXF8738
DAOM242699
DAOM242705
DAOM242701
EXF8749
DAOM242697
DAOM242702
DAOM242700
EXF8754
CBS136849
CBS136836
DAOM242704
EXF8747
DAOM242698
DAOM242696
DAOM242800
EXF8741
DAOM242694
EXF8758
CBS633.66
DAOM242799
EXF8740
CBS136842
DAOM242703
EXF8745
EXF8757
CBS136837
CBS136838
CBS112378
DAOM242695
CBS136827
EXF8592
CBS136848
EXF8351
EXF5917
EXF8344
EXF8342
CBS136829
CBS136846
EXF8315
EXF8327
EXF8353
EXF8343
EXF8314
EXF8313
EXF8352
CBS136831
EXF8356
CBS136833
EXF5916
EXF8590
EXF8355
EXF5914
EXF8591
CBS136839
CBS136840
CBS110599
EXF8341
EXF8586
EXF5919
EXF8587
CBS136834
CBS136832
CBS116628
CBS110624
EXF5915
EXF8345
CBS136844
CBS136835
EXF8299
EXF8588
EXF8354
CBS136830

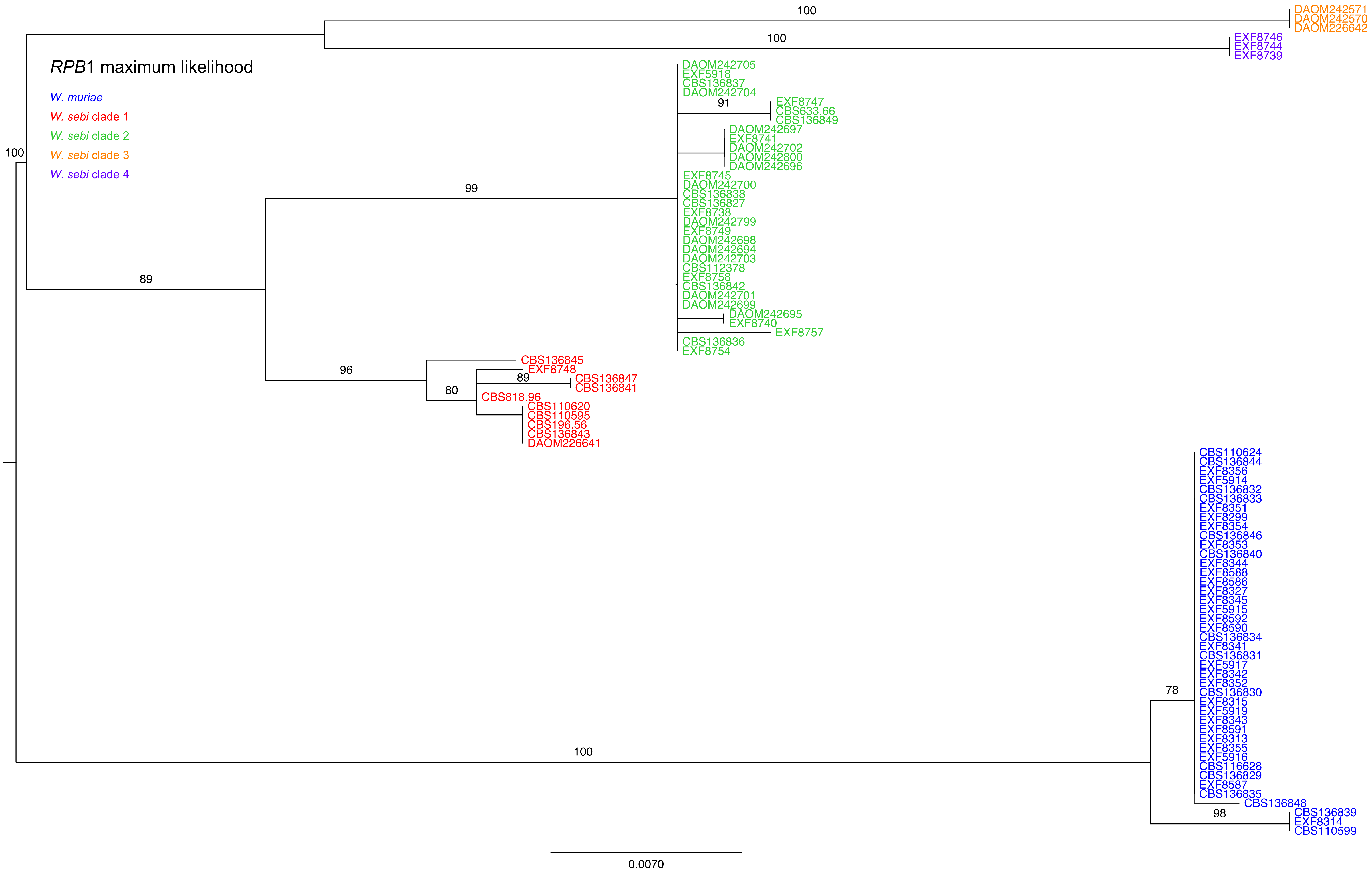
RPB1 maximum likelihood
W. muriae
W. sebi clade 1
W. sebi clade 2
W. sebi clade 3
W. sebi clade 4
DAOM242571
DAOM242570
DAOM226642
EXF8746
EXF8744
EXF8739
DAOM242705
EXF5918
CBS136837
DAOM242704
EXF8747
CBS633.66
CBS136849
DAOM242697
EXF8741
DAOM242702
DAOM242800
DAOM242696
EXF8745
DAOM242700
CBS136838
CBS136827
EXF8738
DAOM242799
EXF8749
DAOM242698
DAOM242694
DAOM242703
CBS112378
EXF8758
CBS136842
DAOM242701
DAOM242699
DAOM242695
EXF8740
EXF8757
CBS136836
EXF8754
CBS136845
EXF8748
CBS136847
CBS136841
CBS818.96
CBS110620
CBS110595
CBS196.56
CBS136843
DAOM226641
CBS110624
CBS136844
EXF8356
EXF5914
CBS136832
CBS136833
EXF8351
EXF8299
EXF8354
CBS136846
EXF8353
CBS136840
EXF8344
EXF8588
EXF8586
EXF8327
EXF8345
EXF5915
EXF8592
EXF8590
CBS136834
EXF8341
CBS136831
EXF5917
EXF8342
EXF8352
CBS136830
EXF8315
EXF5919
EXF8343
EXF8591
EXF8313
EXF8355
EXF5916
CBS116628
CBS136829
EXF8587
CBS136835
CBS136848
CBS136839
EXF8314
CBS110599
0.0070

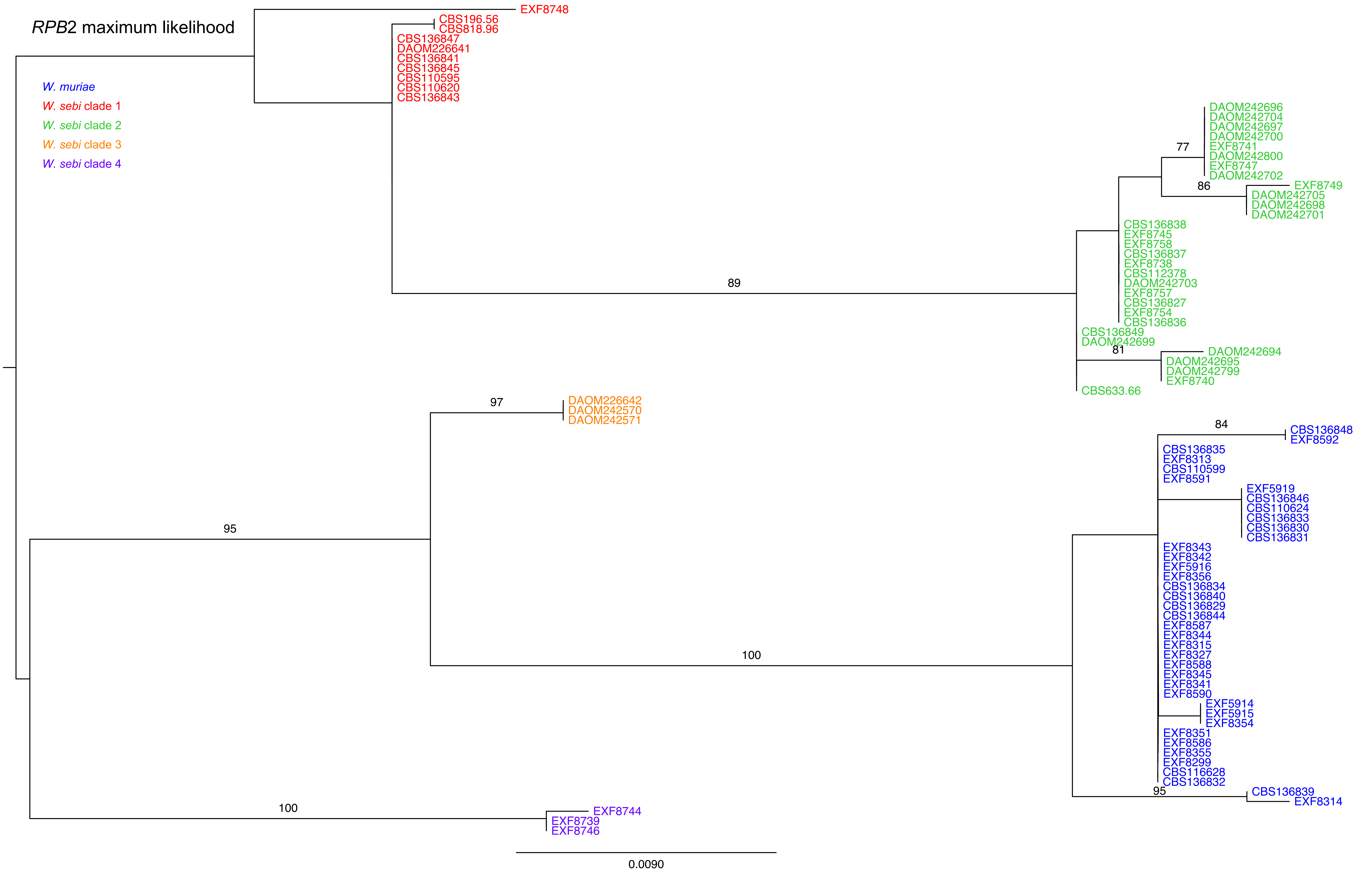

RPB2 maximum likelihood
W. muriae
W. sebi clade 1
W. sebi clade 2
W. sebi clade 3
W. sebi clade 4
EXF8748
CBS196.56
CBS818.96
CBS136847
DAOM226641
CBS136841
CBS136845
CBS110595
CBS110620
CBS136843
DAOM242696
DAOM242704
DAOM242697
DAOM242700
EXF8741
DAOM242800
EXF8747
DAOM242702
77
86
EXF8749
DAOM242705
DAOM242698
DAOM242701
CBS136838
EXF8745
EXF8758
CBS136837
EXF8738
CBS112378
DAOM242703
EXF8757
CBS136827
EXF8754
CBS136836
89
CBS136849
DAOM242699
81
DAOM242694
DAOM242695
DAOM242799
EXF8740
CBS633.66
97
DAOM226642
DAOM242570
DAOM242571
84
CBS136848
EXF8592
CBS136835
EXF8313
CBS110599
EXF8591
EXF5919
CBS136846
CBS110624
CBS136833
CBS136830
CBS136831
95
EXF8343
EXF8342
EXF5916
EXF8356
CBS136834
CBS136840
CBS136829
CBS136844
EXF8587
EXF8344
EXF8315
EXF8327
EXF8588
EXF8345
EXF8341
EXF8590
100
EXF5914
EXF5915
EXF8354
EXF8351
EXF8586
EXF8355
EXF8299
CBS116628
CBS136832
95
CBS136839
EXF8314
100
EXF8744
EXF8739
EXF8746
0.0090

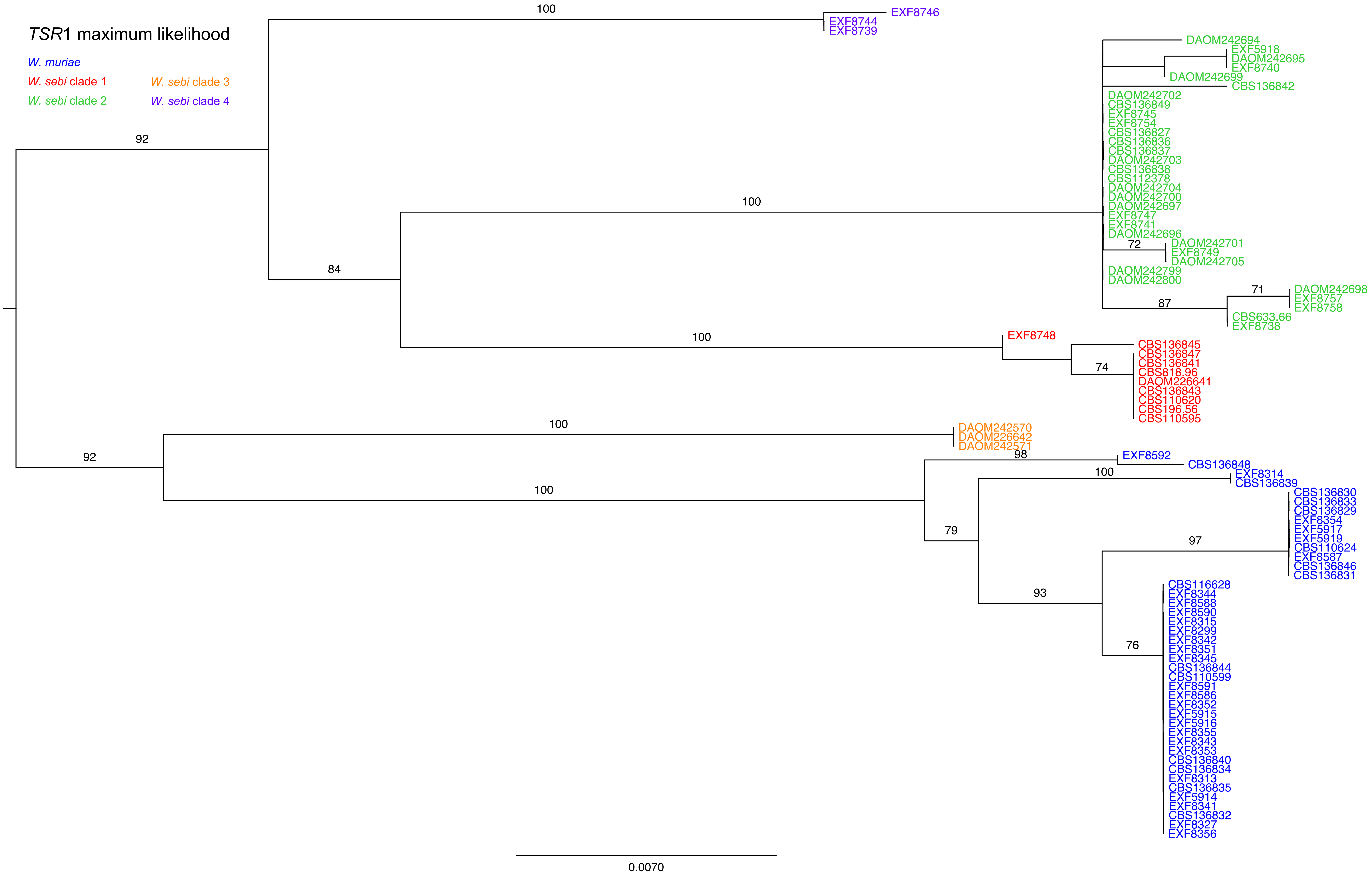
TSR1 maximum likelihood
W. muriae
W. sebi clade 1
W. sebi clade 2
W. sebi clade 3
W. sebi clade 4
100
EXF8746
EXF8744
EXF8739
DAOM242694
EXF5918
DAOM242695
EXF8740
DAOM242699
CBS136842
DAOM242702
CBS136849
EXF8745
EXF8754
CBS136827
CBS136836
CBS136837
DAOM242703
CBS136838
CBS112378
DAOM242704
DAOM242700
DAOM242697
EXF8747
EXF8741
DAOM242696
72
DAOM242701
EXF8749
DAOM242705
DAOM242799
DAOM242800
92
100
84
71
DAOM242698
EXF8757
EXF8758
87
CBS633.66
EXF8738
100
EXF8748
CBS136845
CBS136847
CBS136841
74
CBS818.96
DAOM226641
CBS136843
CBS110620
CBS196.56
CBS110595
100
DAOM242570
DAOM226642
DAOM242571
98
EXF8592
CBS136848
92
100
EXF8314
CBS136839
100
CBS136830
CBS136833
CBS136829
EXF8354
EXF5917
EXF5919
CBS110624
EXF8587
CBS136846
CBS136831
79
97
93
CBS116628
EXF8344
EXF8588
EXF8590
EXF8315
EXF8299
EXF8342
EXF8351
EXF8345
CBS136844
CBS110599
EXF8591
EXF8586
EXF8352
EXF5915
EXF5916
EXF8355
EXF8343
EXF8353
CBS136840
CBS136834
EXF8313
CBS136835
EXF5914
EXF8341
CBS136832
EXF8327
EXF8356
76
0.0070

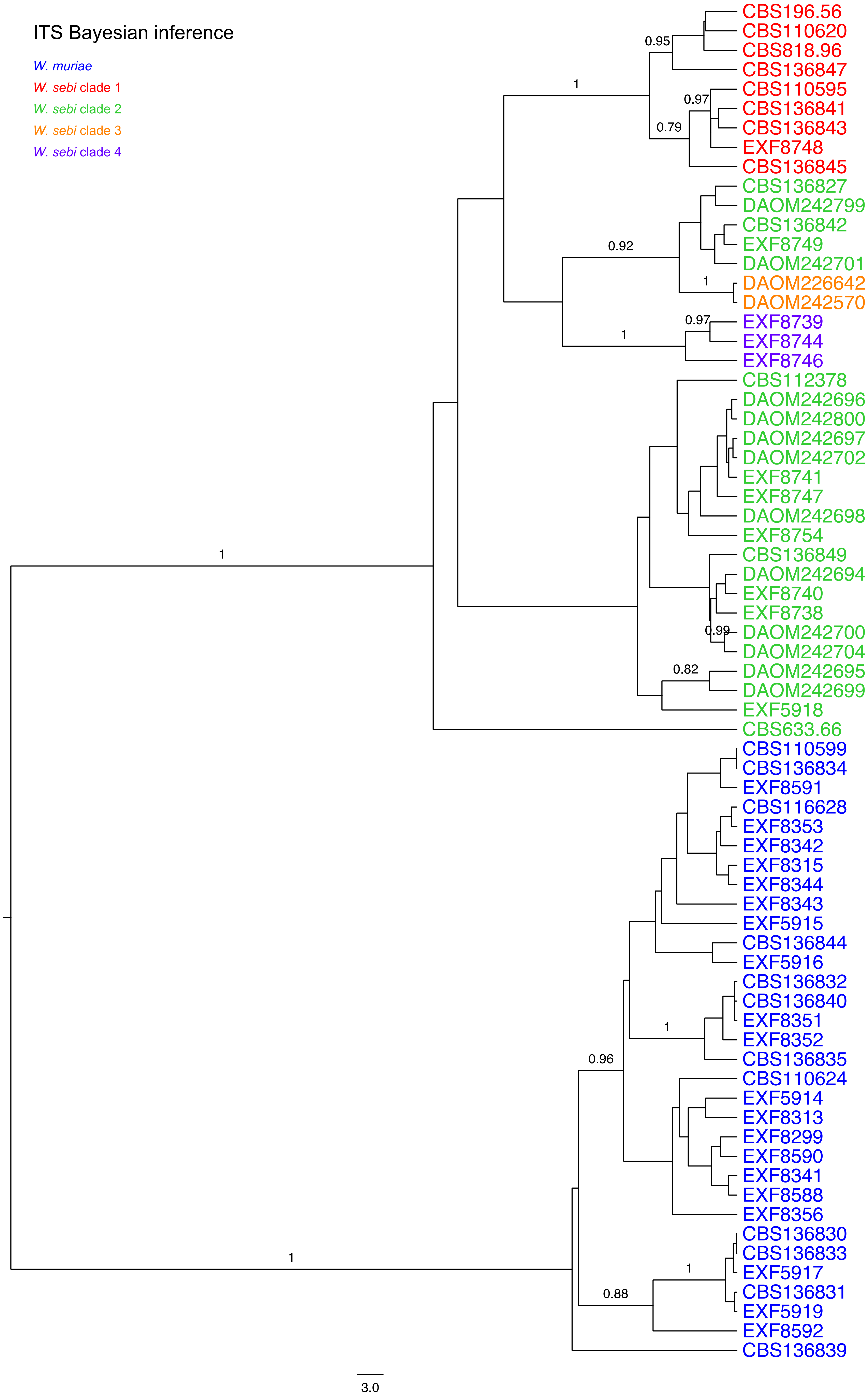

ITS Bayesian inference
W. muriae
W. sebi clade 1
W. sebi clade 2
W. sebi clade 3
W. sebi clade 4
CBS196.56
CBS110620
CBS818.96
CBS136847
CBS110595
CBS136841
CBS136843
EXF8748
CBS136845
CBS136827
DAOM242799
CBS136842
EXF8749
DAOM242701
DAOM226642
DAOM242570
EXF8739
EXF8744
EXF8746
CBS112378
DAOM242696
DAOM242800
DAOM242697
DAOM242702
EXF8741
EXF8747
DAOM242698
EXF8754
CBS136849
DAOM242694
EXF8740
EXF8738
DAOM242700
DAOM242704
DAOM242695
DAOM242699
EXF5918
CBS633.66
CBS110599
CBS136834
EXF8591
CBS116628
EXF8353
EXF8342
EXF8315
EXF8344
EXF8343
EXF5915
CBS136844
EXF5916
CBS136832
CBS136840
EXF8351
EXF8352
CBS136835
CBS110624
EXF5914
EXF8313
EXF8299
EXF8590
EXF8341
EXF8588
EXF8356
CBS136830
CBS136833
EXF5917
CBS136831
EXF5919
EXF8592
CBS136839
0.95
1
0.97
0.79
0.92
1
0.97
1
1
0.99
0.82
1
0.96
1
0.88
1
3.0

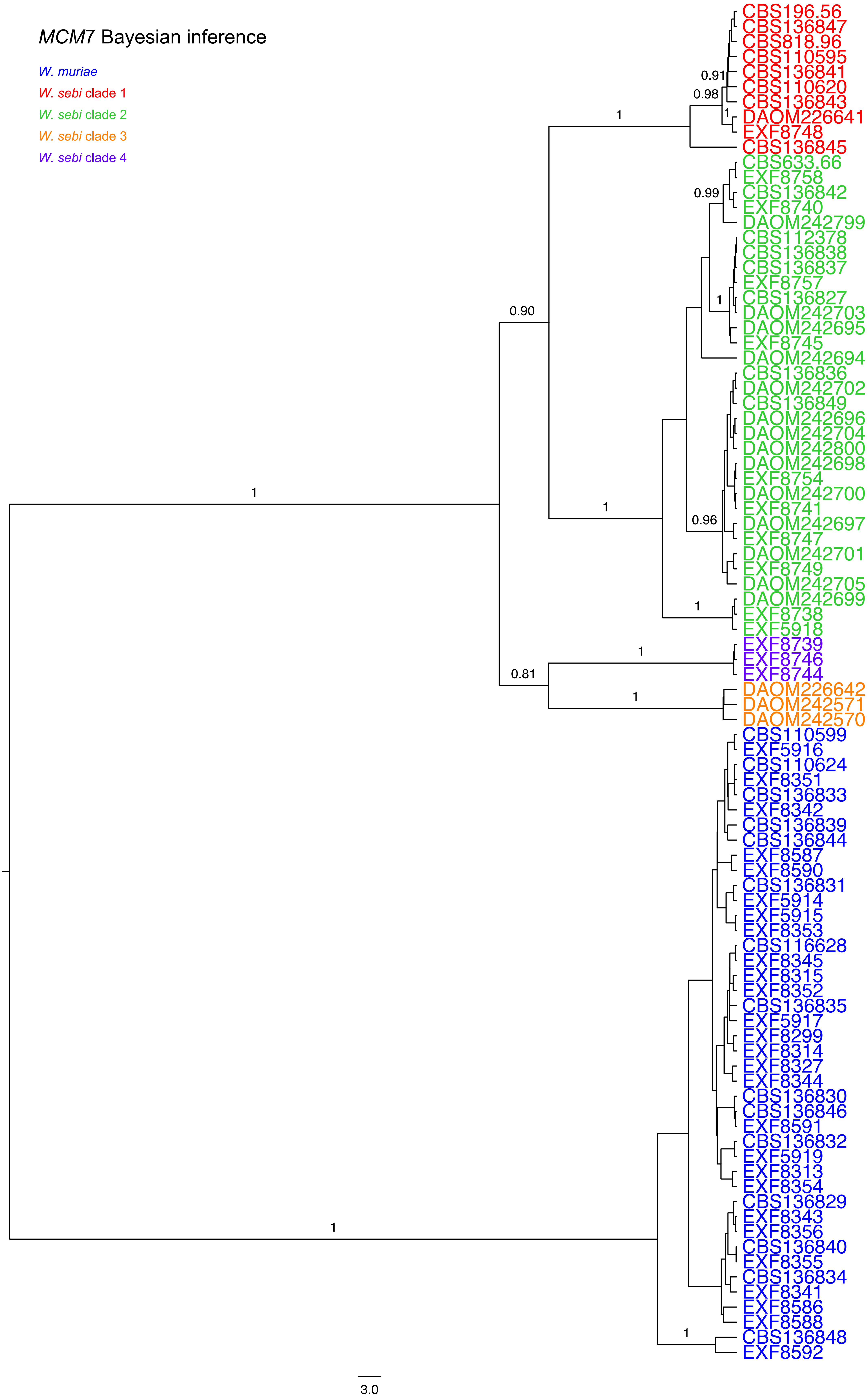
MCM7 Bayesian inference
W. muriae
W. sebi clade 1
W. sebi clade 2
W. sebi clade 3
W. sebi clade 4
CBS196.56
CBS136847
CBS818.96
CBS110595
CBS136841
CBS110620
CBS136843
DAOM226641
EXF8748
CBS136845
CBS633.66
EXF8758
CBS136842
EXF8740
DAOM242799
CBS112378
CBS136838
CBS136837
EXF8757
CBS136827
DAOM242703
DAOM242695
EXF8745
DAOM242694
CBS136836
DAOM242702
CBS136849
DAOM242696
DAOM242704
DAOM242800
DAOM242698
EXF8754
DAOM242700
EXF8741
DAOM242697
EXF8747
DAOM242701
EXF8749
DAOM242705
DAOM242699
EXF8738
EXF5918
EXF8739
EXF8746
EXF8744
DAOM226642
DAOM242571
DAOM242570
CBS110599
EXF5916
CBS110624
EXF8351
CBS136833
EXF8342
CBS136839
CBS136844
EXF8587
EXF8590
CBS136831
EXF5914
EXF5915
EXF8353
CBS116628
EXF8345
EXF8315
EXF8352
CBS136835
EXF5917
EXF8299
EXF8314
EXF8327
EXF8344
CBS136830
CBS136846
EXF8591
CBS136832
EXF5919
EXF8313
EXF8354
CBS136829
EXF8343
EXF8356
CBS136840
EXF8355
CBS136834
EXF8341
EXF8586
EXF8588
CBS136848
EXF8592
3.0

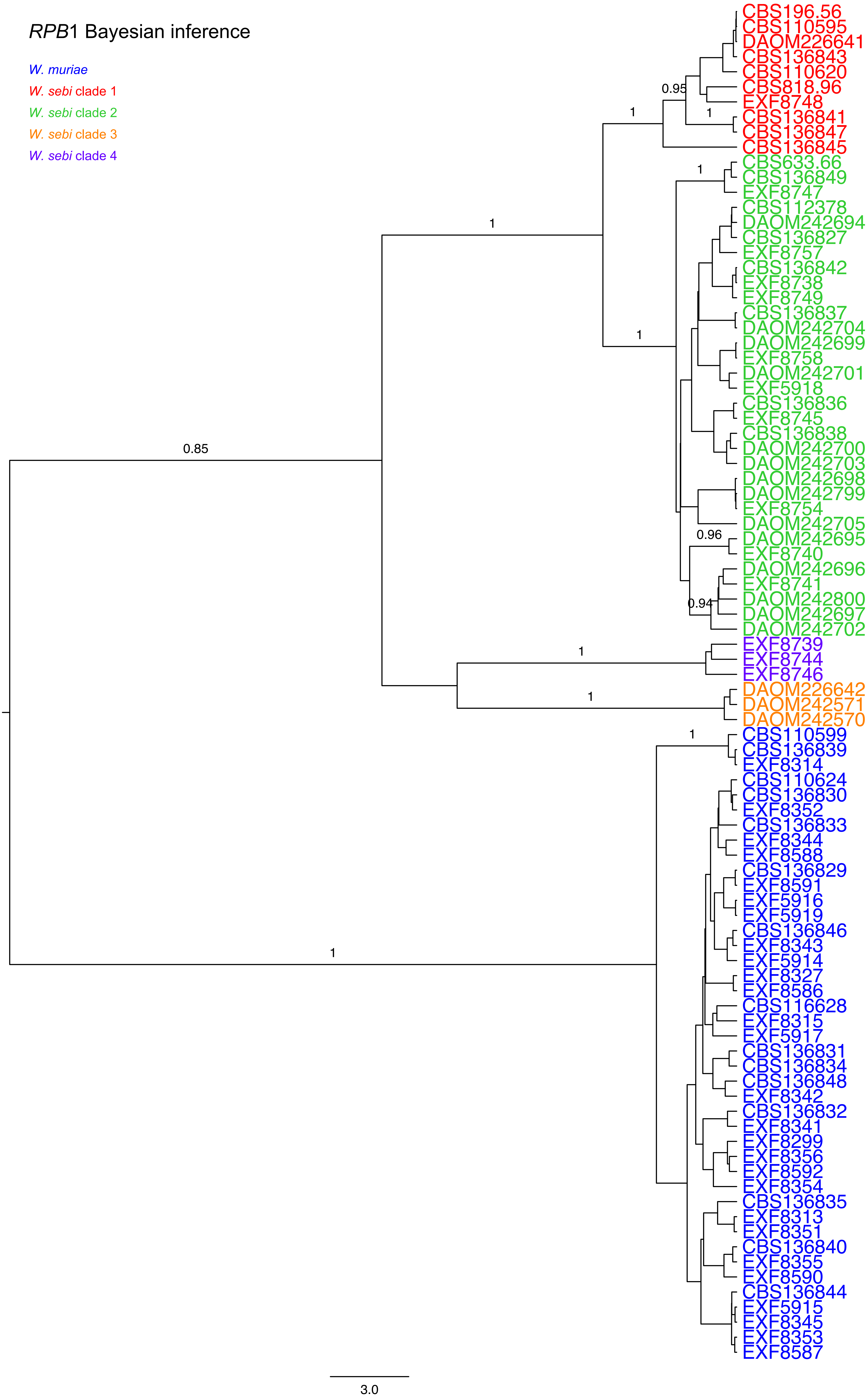
RPB1 Bayesian inference
W. muriae
W. sebi clade 1
W. sebi clade 2
W. sebi clade 3
W. sebi clade 4
CBS196.56
CBS110595
DAOM226641
CBS136843
CBS110620
CBS818.96
EXF8748
CBS136841
CBS136847
CBS136845
CBS633.66
CBS136849
EXF8747
CBS112378
DAOM242694
CBS136827
EXF8757
CBS136842
EXF8738
EXF8749
CBS136837
DAOM242704
DAOM242699
EXF8758
DAOM242701
EXF5918
CBS136836
EXF8745
CBS136838
DAOM242700
DAOM242703
DAOM242698
DAOM242799
EXF8754
DAOM242705
DAOM242695
EXF8740
DAOM242696
EXF8741
DAOM242800
DAOM242697
DAOM242702
EXF8739
EXF8744
EXF8746
DAOM226642
DAOM242571
DAOM242570
CBS110599
CBS136839
EXF8314
CBS110624
CBS136830
EXF8352
CBS136833
EXF8344
EXF8588
CBS136829
EXF8591
EXF5916
EXF5919
CBS136846
EXF8343
EXF5914
EXF8327
EXF8586
CBS116628
EXF8315
EXF5917
CBS136831
CBS136834
CBS136848
EXF8342
CBS136832
EXF8341
EXF8299
EXF8356
EXF8592
EXF8354
CBS136835
EXF8313
EXF8351
CBS136840
EXF8355
EXF8590
CBS136844
EXF5915
EXF8345
EXF8353
EXF8587
0.95
1
1
1
1
1
0.96
0.94
0.85
1
1
1
1
3.0

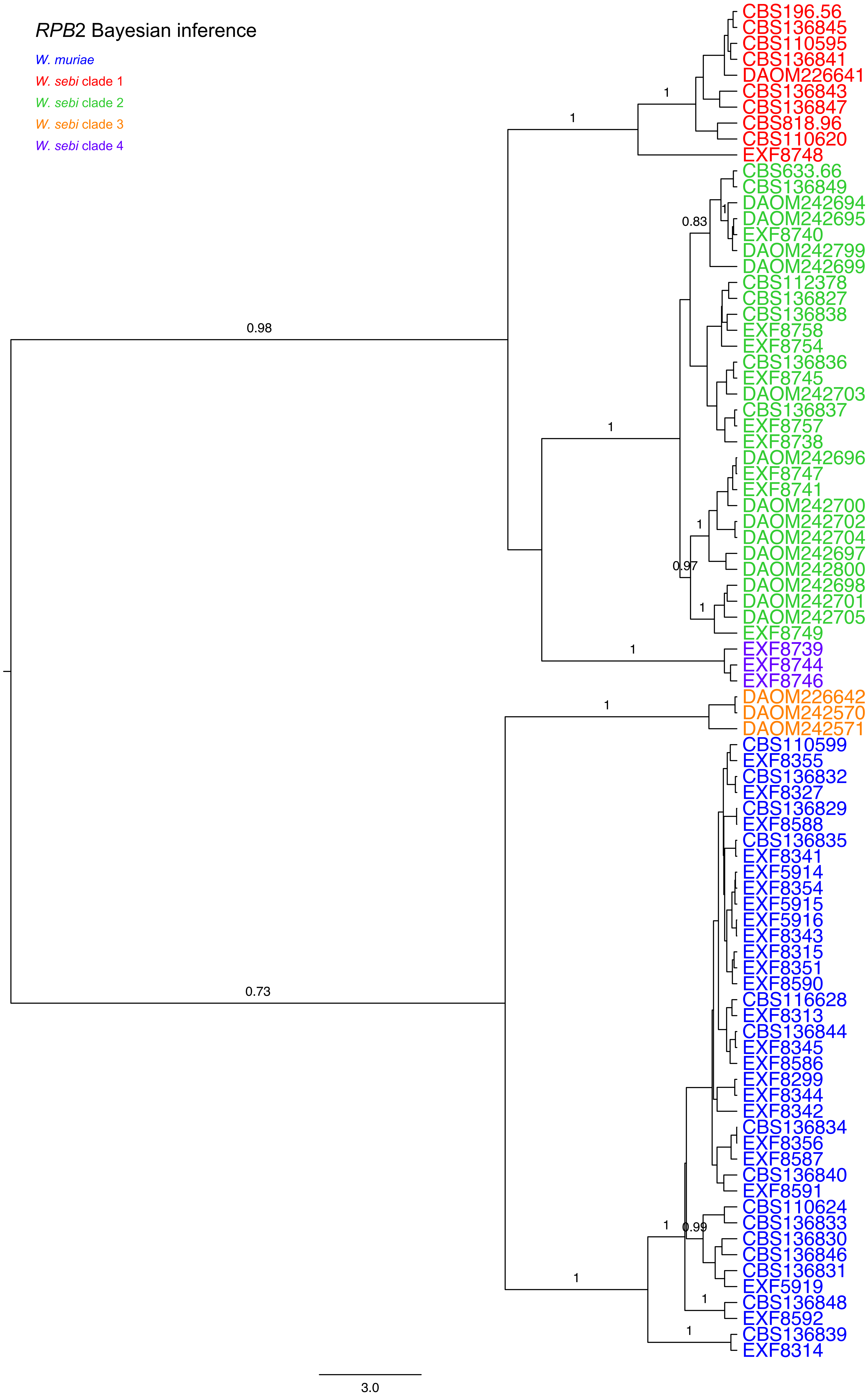

RPB2 Bayesian inference
W. muriae
W. sebi clade 1
W. sebi clade 2
W. sebi clade 3
W. sebi clade 4
CBS196.56
CBS136845
CBS110595
CBS136841
DAOM226641
CBS136843
CBS136847
CBS818.96
CBS110620
EXF8748
CBS633.66
CBS136849
DAOM242694
DAOM242695
EXF8740
DAOM242799
DAOM242699
CBS112378
CBS136827
CBS136838
EXF8758
EXF8754
CBS136836
EXF8745
DAOM242703
CBS136837
EXF8757
EXF8738
DAOM242696
EXF8747
EXF8741
DAOM242700
DAOM242702
DAOM242704
DAOM242697
DAOM242800
DAOM242698
DAOM242701
DAOM242705
EXF8749
EXF8739
EXF8744
EXF8746
DAOM226642
DAOM242570
DAOM242571
CBS110599
EXF8355
CBS136832
EXF8327
CBS136829
EXF8588
CBS136835
EXF8341
EXF5914
EXF8354
EXF5915
EXF5916
EXF8343
EXF8315
EXF8351
EXF8590
CBS116628
EXF8313
CBS136844
EXF8345
EXF8586
EXF8299
EXF8344
EXF8342
CBS136834
EXF8356
EXF8587
CBS136840
EXF8591
CBS110624
CBS136833
CBS136830
CBS136846
CBS136831
EXF5919
CBS136848
EXF8592
CBS136839
EXF8314
0.98
0.73
0.83
0.97
0.99
3.0

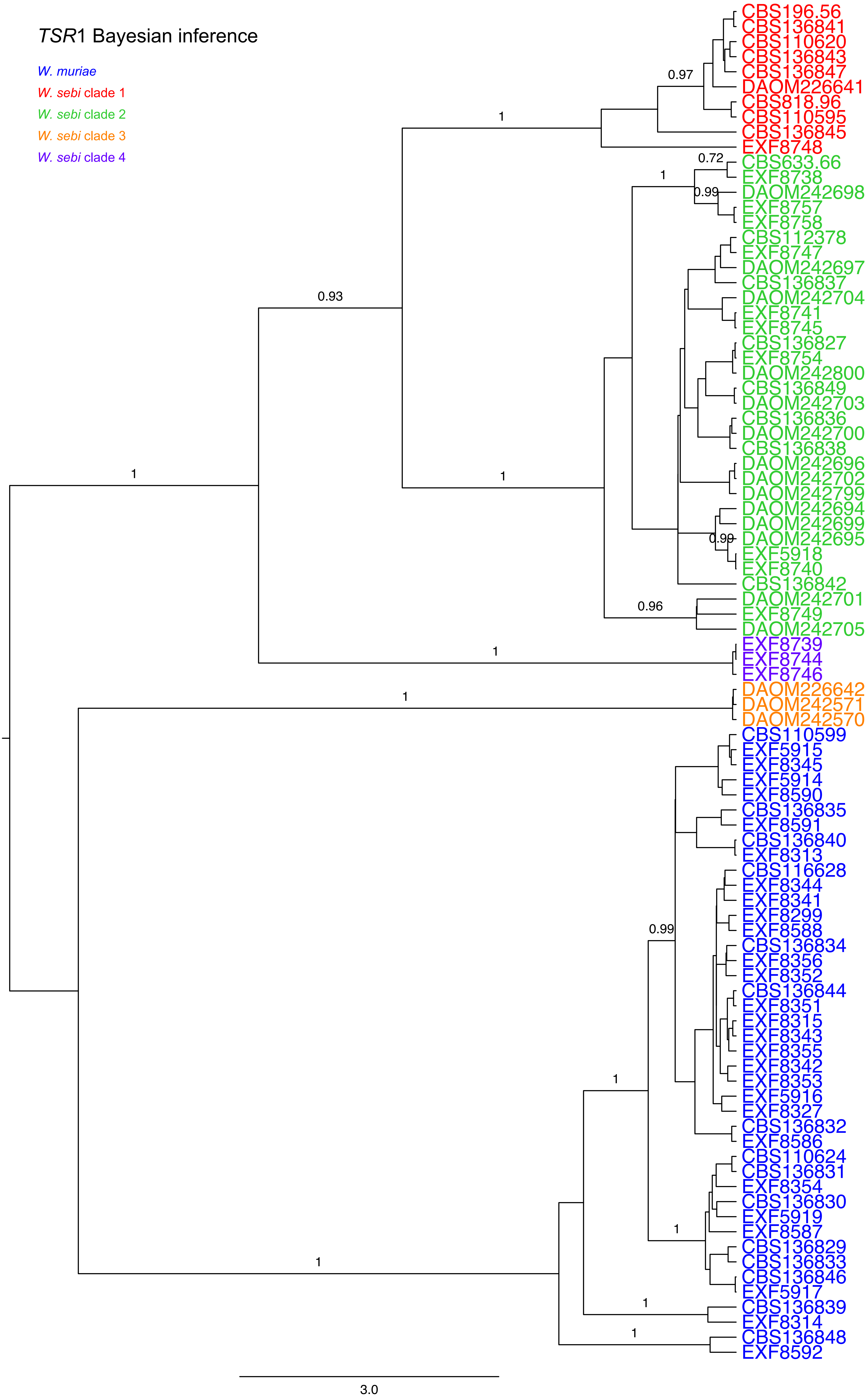

*TSR*1 Bayesian inference
W. muriae
W. sebi clade 1
W. sebi clade 2
W. sebi clade 3
W. sebi clade 4
CBS196.56
CBS136841
CBS110620
CBS136843
CBS136847
DAOM226641
CBS818.96
CBS110595
CBS136845
EXF8748
CBS633.66
EXF8738
DAOM242698
EXF8757
EXF8758
CBS112378
EXF8747
DAOM242697
CBS136837
DAOM242704
EXF8741
EXF8745
CBS136827
EXF8754
DAOM242800
CBS136849
DAOM242703
CBS136836
DAOM242700
CBS136838
DAOM242696
DAOM242702
DAOM242799
DAOM242694
DAOM242699
DAOM242695
EXF5918
EXF8740
CBS136842
DAOM242701
EXF8749
DAOM242705
EXF8739
EXF8744
EXF8746
DAOM226642
DAOM242571
DAOM242570
CBS110599
EXF5915
EXF8345
EXF5914
EXF8590
CBS136835
EXF8591
CBS136840
EXF8313
CBS116628
EXF8344
EXF8341
EXF8299
EXF8588
CBS136834
EXF8356
EXF8352
CBS136844
EXF8351
EXF8315
EXF8343
EXF8355
EXF8342
EXF8353
EXF5916
EXF8327
CBS136832
EXF8586
CBS110624
CBS136831
EXF8354
CBS136830
EXF5919
EXF8587
CBS136829
CBS136833
CBS136846
EXF5917
CBS136839
EXF8314
CBS136848
EXF8592
3.0
